# Subgroup differences in public attitudes, preferences and self-reported behaviour related to deceased organ donation before and after the introduction of the ‘soft’ opt-out consent system in England: mixed-methods study

**DOI:** 10.1186/s12913-024-11821-3

**Published:** 2024-11-21

**Authors:** Paul Boadu, Leah McLaughlin, Jane Noyes, Stephen O’Neill, Mustafa Al-Haboubi, Lorraine Williams, Jennifer Bostock, Nicholas Mays

**Affiliations:** 1 Policy Innovation and Evaluation Research Unit, Department of Health Services Research and Policy, London School of Hygiene & Tropical Medicine, London, UK; 2 School of Medical and Health Sciences, Bangor University, Bangor, UK

**Keywords:** Organ donation, Deemed consent, Inequalities, Latent class analysis, Mixed-methods, Public attitudes and behaviours, Patient and public involvement and engagement

## Abstract

**Background:**

In the UK, over 7,000 people are on the waiting list for an organ transplant and there are inequalities in need, access and waiting time for organs, with notable differences between minority ethnic groups. In May 2020, England changed the law and introduced a ‘soft’ opt-out system of consent to organ donation with a view to increase consent rates. We aimed to learn more about the impact of the law change on attitudes and views likely to be relevant to consent to deceased organ donation between different population subgroups.

**Methods:**

Mixed-methods design involving latent class analysis of data from twelve repeated cross-sectional surveys undertaken from 2015 to 2021 (*n* = 19,011); analysis of the law change survey dataset collected quarterly from 2018 to 2022 (*n* = 45,439); and interviews with purposively selected members of the public (*n* = 30) with a focus on minority perspectives.

**Results:**

Support for the principle of deceased organ donation remained high and stable in the general population (80%) but was 20% lower among ethnic minorities. From 2018 to 2022, an average of 58% of the general population was aware of the law change; this was lower among minority ethnic groups (31%). We identified four population subgroups (supportive donors (24% of the population); unengaged donors (22%); uncommitted donors (46%); and unsupportive donors (9%)). Interview themes included the challenges of discussing organ donation decisions, balancing autonomy with respecting family relationships, targeted misinformation, frustrations at the lack of consensus between community leaders, limited understanding of what happens during the end-of-life care leading to organ donation, and how this aligns with cultural values and preferences.

**Conclusion:**

Implementation of the law change has not been associated to date with any change in public attitudes and preferences likely to influence consent overall or in minority ethnic groups in England. Uncommitted donors may benefit from encouragement to express their organ donation decision, and unengaged donors from attempts to address mis/information, confusion, and uncertainty. Interventions to raise the consent rate need to take account of the significant role of the family as well as wider community influences on attitudes, preferences and decision-making, particularly among certain minority (ethnic) groups.

**Supplementary Information:**

The online version contains supplementary material available at 10.1186/s12913-024-11821-3.

## Background

Compared to other forms of treatment, organ transplantation is the most cost-effective, and often the only life-saving treatment for people with end-stage organ failure [1]. In the UK, over 7,000 people are on the transplant waiting list with about three people estimated to die daily while waiting for a transplant [2]. Despite the fact that in countries with developed healthcare systems support for organ donation in principle is high, one of the major challenges facing deceased organ donation is the low consent rate in practice. The average consent rate in 2020 in the UK for deceased organ donation was 67.2% with marked differences between ethnic groups. The families of ethnically white donors were 1.7 times more likely to give consent (70.5%) compared with families of donors from other ethnic groups (defined as all non-white donors) (41.7%) [3].

Several factors have been identified to influence consent rate for deceased organ donation in different population subgroups. They include organization of the healthcare system and the level of public trust in the healthcare system [4]; knowledge about the patient’s wishes, involvement of a specialist nurse [5]; differences in how families make sense of donation decisions, emotional attitudes towards the dead body and general acceptability of deceased donation [6–9]; public awareness of the consent model in place and the role relatives play in the decision making process [4]; and socioeconomic status, demographic factors, religious and cultural beliefs [10–13]. Combined, these complex and interrelated factors with regard to consent can contribute to reducing the number of organs available for transplantation. Ethnicity is also important for matching organs as there are benefits to receiving an organ from a person with a similar ethnic background [14]. As a consequence, there are ethnic inequalities in the UK related to organ donation and transplantation with people from an ethnic minority background having a greater need for transplantation but a lower donation rate [15–17].

To increase the consent rate and availability of deceased organs for transplantation, many countries have introduced versions of ‘opt-out’ systems of consent to deceased organ donation. In May 2020, England implemented a ‘soft’ opt-out system of consent to deceased organ donation. This legislative change adopts the principle of deemed consent – implying that if no active donor decision (opt-out or opt-in) has been expressed either verbally or on the National Health Service (NHS) Blood and Transplant (NHSBT) Organ Donor Register, individuals (who meet the specific eligibility criteria) are deemed to have consented to organ donation after they die. NHSBT is a NHS specialist agency responsible for managing the donation, storage, and transplantation of blood and blood components, organs, tissues, bone marrow and stem cells, and researching new treatments and processes in England and across the UK. The purpose of the law change was to switch the default position of citizens to one that supports organ donation with the hope that this would increase consent rates. The law is, however, ‘soft’ meaning that families can still, in practice, override the deceased person’s decision made in life. This was an intentional addition to avoid criticisms and a potential public backlash if the government were seen to be ‘taking organs’ without consent. An overview of the implementation of the ‘soft’ opt-out into the previous opt-in system in England is provided in Supplementary file 1.

The NHSBT Organ Donor Register was established in 1994 – a national database where residents in the UK can record a decision about becoming an organ donor. Over time, additional ‘nudges’ were added signposting people to the register (e.g., via issuing of drivers’ licences, and shopping points cards at retail outlets (e.g. Boots pharmacy chain), as well as through local and national media campaigns). In 2015, Wales (a country within the UK with devolved responsibility for the NHS) implemented a ‘soft’ opt-out system of organ donation. A key part of the policy package was updating the NHSBT Organ Donor Register to offer citizens the option to opt out of organ donation in addition to giving more say to people about which organs and tissues they wanted to donate after they died. It was not possible to alter the organ donor register exclusively for Wales, so, in April 2015 (eight months before the law change was implemented in Wales), the NHSBT Organ Donor Register (covering Wales, England, Scotland and Northern Ireland) was adapted to align with these options. In 2019, the UK Parliament voted to switch to a similar ‘soft’ opt-out system in England, and, as part of this package of change, the register was altered to allow people to include details of their faith and ethnicity. The full list of options and details currently available on the NHSBT Organ Donor Register is provided in Supplemental file 2. Despite an increasing number of countries switching to opt-out systems, evidence is mixed as to public awareness of such systems and whether they have a positive influence on consent to deceased organ donation [18].

## The study

This study aimed to assess the English public’s knowledge, attitudes, reported behaviour and preferences towards deceased organ donation, and to learn more about the potential impact of the law change on public attitudes, preferences and self-reported behaviour likely to be relevant to consent to deceased organ donation. We sought to answer the following research questions:

1. What is the level of awareness of organ donation publicity highlighting the change in the role of families in deceased organ donation decisions as a result of the law change?
2. Has the law change been associated with any changes in public support, reported behaviour, attitudes, and willingness to donate deceased organs since its implementation?
3. What are the barriers to deceased organ donation reported among the public in England?
4. Are there population subgroups with different preferences towards deceased organ donation in England, and may they benefit potentially from targeted policy interventions to encourage support for donation?

### Theoretical perspective

We applied the theory of rational choice as a lens to help interpret and integrate the data (Supplemental file 3). This theory assumes that individuals are rational and rely on information, reasoning and logic to make choices and decisions that give them the highest satisfaction [19, 20]. In this context, rationality is defined broadly to include the notion of ‘bounded rationality’ which recognises that humans have limited cognitive ability when faced with the range and complexity of information required to take rational decisions and that this constrains their problem-solving capability; that people sometimes make choices that are not in their long-run best interests and that humans are sometimes willing to sacrifice their own interest to help others [21–23]. The choice an individual makes to serve their own best interest is dependent on their personal preferences and attitudes. For example, one person may decide not to smoke for health reasons. Another person may choose to smoke to relieve his/her stress. Despite the choices being opposite, both individuals are assumed to be making these choices freely to get the best outcome for themselves [24].

Although the law change was a manifestation of a strong value preference by legislators that more people should consent to donation (by shifting the default), the media campaign that publicised the law change was value neutral. People were still able to make informed decisions in their own best interests without any implication of state pressure.

We thus chose rational choice theory when studying the impact of the change in legislation, since the legislation is built on the twin assumptions that people make their own rational organ donation decisions and that the change in the default would make it more likely that these decisions would tend towards consent to deceased donation. In addition, the assumption was that individuals would make their own decision using verified and up-to-date sources of information.

The 2019 legislation provided a number of different ways in which people could be seen to have consented to deceased organ donation, both active and passive – by registering on the organ donor register, by telling people who would be in a position to convey their decision to healthcare professionals when they died, or by ‘deemed consent’ given that the law now permits the presumption of consent in circumstances where the individual uses neither of the two previous courses of action.

## Methods

### Study design

We undertook a mixed-methods study involving analysis of both qualitative and quantitative data that were collected independently, analysed separately initially, and then brought together through mapping key findings onto the theoretical framework and developing an integrated narrative summary. The qualitative interview findings were also used to help explain some of the inter-ethnic group differences in findings from the latent class analysis of public survey data.

### Data collection

#### Quantitative data

NHSBT’s national organ donation public survey data for England were shared with the research team. Data comprised (i) an Organ Donation Attitudinal Tracker Survey dataset of twelve repeated cross-sectional surveys collected, roughly eight months apart, from August 2015 to October 2022, and (ii) a Law Change Survey dataset of 32 waves of repeated cross-sectional data collected quarterly from 2018 to 2022 to gauge the level of public awareness of dedicated campaigns to inform the general public about the law change. Of note, the implementation of the media campaign was stopped in March 2020 due to COVID-19 and a second post-implementation media campaign, entitled ‘Leave Them Certain’ was phased in from 2022.

The survey participants were recruited from Kantar’s [25] online panel consisting of approximately 30,000 adults aged 16 years and over who have consented to take part in a range of surveys. Survey participants were recruited by quota sampling with random locational sample selection. Each quota was set based on national Census data on age, education, and geographical region. Different quota were set for each survey to represent the changing population structure. Respondents were invited by email to answer the survey online. The survey questions were in English. Respondents were offered small financial rewards to complete the survey. The samples were weighted to be representative in terms of age, ethnicity and social class of the adult population of England aged 16 years and above.

The questionnaire for the Organ Donation Attitudinal Tracker Survey included questions that elicited respondents’ choices regarding their willingness to donate organs after death (Table 1).

**Table 1:**
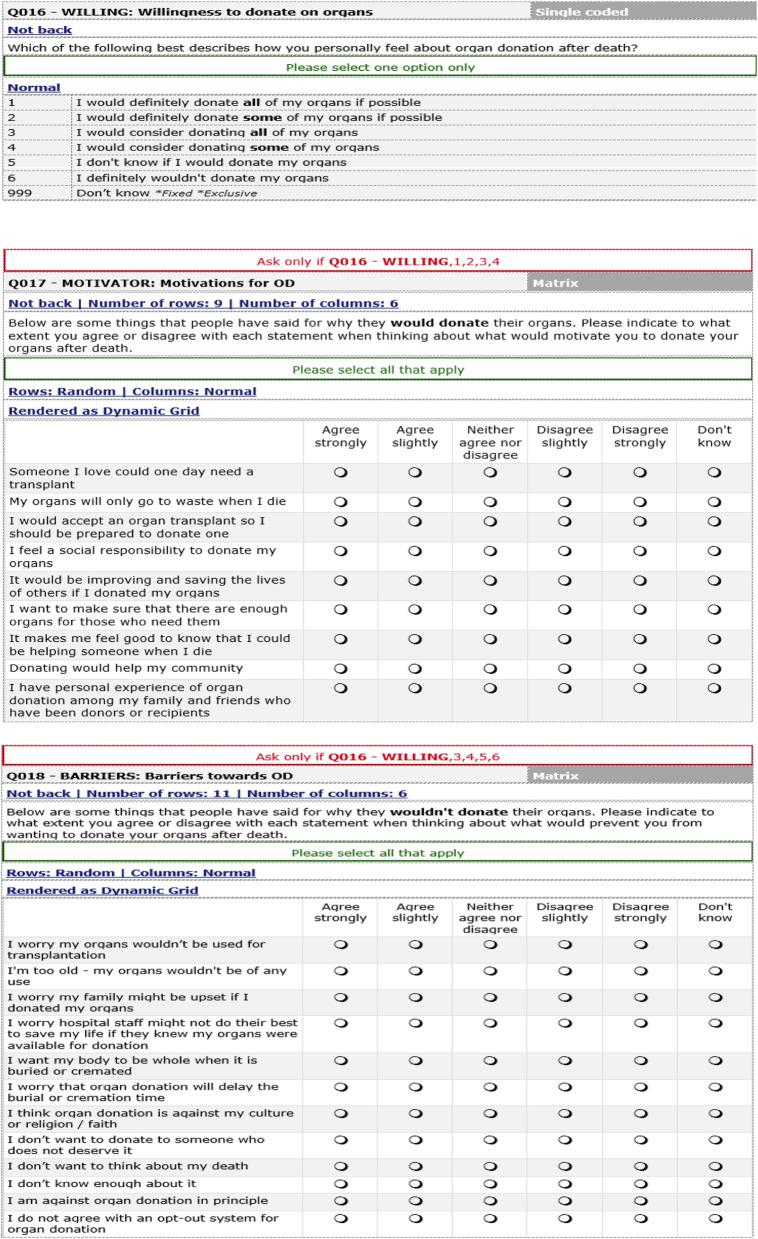
Key questions for the latent class analysis

We excluded all respondents who did not provide their ages (*n* = 284) and those who did not answer the question about their willingness to donate organs after death (*n* = 552). The total sample for each of the Attitudinal Tracker Survey waves used in the analysis ranged from 997 to 2151 with an average sample of 1,710 over the survey period (Supplementary Table 1). The total sample for each of the survey waves used in the analysis of the law change data ranged from 1,261 to 2,556 with an average sample of 1,420 over the survey period (Supplementary Table 2).

#### Qualitative data

We used the population profile of the NHSBT Organ Donor Register to guide construction of a purposive sample of potential participants to target for interviews. We also focused on groups less represented in previous research, and those groups our patient and public advisory group particularly wanted us to include, including people who had opted out on the NHSBT Organ Donor Register, those not supportive of the change in law, and individuals from particular faith groups and non-white ethnicity. We developed a topic guide asking about their views on organ donation, the law change, NHSBT’s publicity campaign, specific ethnic and/or religious views on organ donation and the impact of COVID-19 (Supplementary file 4).

Recruitment was a mix of convenience and snow balling via our patient and public networks (discussed in the PPIE section below). Interviews were a mix of remote (due to COVID-19 via telephone or Teams/Zoom) and face-to-face, one-to-one, with the exception of three small group interviews, ranged between 50 and 80 min and were undertaken by an experienced researcher with a PhD (LMcL).

### Data analysis

#### Statistical analysis

We used Stata Standard Edition version 18. Frequency distributions, weighted percentages, means, and standard deviations were used to describe the characteristics of respondents for the organ donation attitudinal tracker survey and the law change survey. Chi-squared statistics and *p*-values were generated to describe the association between respondents’ characteristics and their organ donation preferences. Due to limitations in data, minority ethnic groups in this analysis refer to all respondents who self-described as having a non-white ethnic background.

Using a stated preference technique [26], and assuming respondents had freely made choices from the options presented to them as shown in Table 1 regarding organ donation, we modelled individuals’ preferences for deceased organ donation subject to their level of motivation or barriers (demotivating factors) to deceased organ donation using data from the attitudinal tracker survey. The motivating factors included altruism (e.g., saving lives, the good feeling that other lives could be helped after death, etc.), benefits (e.g., a loved one could benefit, avoidance of waste, seeing the need to donate based on being willing to receive a transplant, personal experience among family and friends), and social (e.g. a sense of social responsibility, the view that donating will help the community). The demotivating factors included psychological factors (e.g. personal decisions such as wanting the body to be whole when buried or cremated, emotional appeal such as not wanting to think about death; presumptions (e.g. I’m too old and my organs will not be of any use); lack of trust (e.g. a concern that hospital staff might not do their best if they knew patients’ organs were available for donation, worry that the donated organs would not be used for transplantation); and cultural factors (e.g. the degree of family support for deceased donation, worry that the family might be upset by deceased organ donation, or that it would be against cultural and religious views) (see Table 1).

A key assumption of the choice options within a stated preferences approach is that the activities of interest (in this case consent to donating organs) can be described by their attributes and that an individual’s evaluation of the options depends on the levels of these attributes. Individuals’ responses to questions related to their motivations to donate their organs, and reasons why they would not donate their organs (see Table 1) were used to generate mean motivation scores and assign them to each of the five choice options in Table 1. We recoded the Likert scale of the motivating factors such that those who selected ‘strongly agree’ to the statements were given a higher score (i.e., agree strongly = 5, agree slightly = 4, neither agree nor disagree = 3, disagree slightly = 2, and disagree strongly = 1). None of the respondents selected the “don’t know” option in either of the motivation and demotivation statements in Table 1. Reversed codes were used in the case of barriers/demotivating factors. Thus, the more an individual was willing to donate deceased organs, the higher their motivation score, and vice versa.

We used a latent class regression model to estimate and identify subgroups of the population that have a similar inclination towards deceased organ donation. The log-likelihood function maximized in the estimation is given as:

1 $$\:logL=\sum\nolimits_{n=1}^{N}\sum\nolimits_{j=1}^{J}{y}_{nj}log\sum\nolimits_{s=1}^{S}\left[\frac{\text{e}\text{x}\text{p}\left({\beta}_{s}^{{ASC}_{j}}+{\beta}_{s}{X}_{nj}\right)}{\sum\nolimits_{h=1}^{J}\text{e}\text{x}\text{p}({\beta}_{s}^{{ASC}_{Jh}}+{\beta}_{s}{X}_{nh})}\right]\:\:\:\:\:\:\:\:\:\:$$

Where:

*J* is the total number of alternative choices, *j*=1,….,6; and *j* denote particular choices among the alternatives.

${Y_{nj}}$ is an indicator for whether individual $\:n$ chooses $\:{j}^{th}$ alternative within the choice set (options). This is equal to 1 (chosen) or 0 (not chosen), and we assume that an individual $\:n$ will choose ($\:j$) in preference to other alternatives ($\:h$*)* if and only if ${U_{nj}}\,>\,{U_{nh}}$, where ${U_{nj}}$ is the level of motivation/demotivation towards deceased organ donation.

*s* identifies a given subgroup among the *S* subgroups (latent classes);

$\beta _{s}^{{AS{C_j}}}$ is a vector of coefficients of the group-specific, alternative-specific constants for alternative *j*.

${X_{nj}}$ is a vector of observed variables including the level of motivation for deceased organ donation, and socio-demographic characteristics; the estimates for their coefficients, ${\beta _s}$, are determined by maximizing the log-likelihood function.

To estimate the model, we first conducted statistical tests using the minimum of the Akaike Information Criterion (AIC), and the Bayesian Information Criterion (BIC) estimates to determine the number of subgroups, *S*, within the population to be included in the model [27–30]. The tests were run on all twelve waves of survey data consecutively (see Table 2, and supplementary Table 4). The results showed a minimum of two subgroups and a maximum of four subgroups within the population with similar inclinations towards deceased organ donation. We therefore chose to present the results from the data set with most diverse population subgroup responses, wave 10, because it provides a spectrum of all subgroups within the population and may be especially useful for designing targeted interventions to support the new systems of consent to deceased organ donation in the UK. The differences in association of the characteristics of individuals belonging to different subgroups of the population were determined using a t-test and Pearson’s $\:{x}^{2}\:$test.

#### Qualitative analysis

Interviews were transcribed verbatim and uploaded into NVIVO version 12 [31]. Thematic analysis was undertaken [32]. After familiarisation through reading field notes and re-reading transcripts, coding was undertaken to identify actions and behaviours following implementation of the law change, motivations to donate or not, media awareness (including ‘nudges’), differences between ethnic minority perspectives, (barriers to) talking about and normalising organ donation as part of end-of-life care and suggestions to promote organ donation. The themes were then shared with a multi-disciplinary team of experts and a range of lay audiences to assist in developing a consensus set of findings. Findings were mapped against the theoretical framework.

### Validity, reliability, and rigour

For the statistical analysis, an additional layer of rigour was applied by comparing our analysis of the survey data to that of NHSBT. We used four quality criteria (credibility, dependability, confirmability, and transferability) to assess the qualitative analysis [33]. For example, interim findings were shared at several meetings with a multi-disciplinary advisory group which had opportunity to comment on the content and advise on ways to address gaps in the data and what might be further strengthened. The research team was also able to present the findings at events specifically focused on inequalities in organ donation to test their plausibility and relevance.

### Reflexivity

The research team comprised of professional and lay researchers with expertise in health and social care, qualitative and quantitative research methods, and experience of previous research into organ donation. Differences in interpretation were resolved through regular team meetings and discussion.

### Patient and public involvement

We developed a wide patient and public advisory group for this study including organisations and individuals representing ethnic minority general health and social issues, bereavement care services, and charities supporting donor families and transplant recipients, live donation, and blood donation. We also had a public member, with experience of organ donation as a carer, as a full member of the research team. This approach facilitated the recruitment of members of the public for interviews, and provided additional contextual information, as well as input into analysis, interpretation, and integration of findings [34, 35].

### Integration of findings using analytical and interpretive framework

**Fig. 1:**
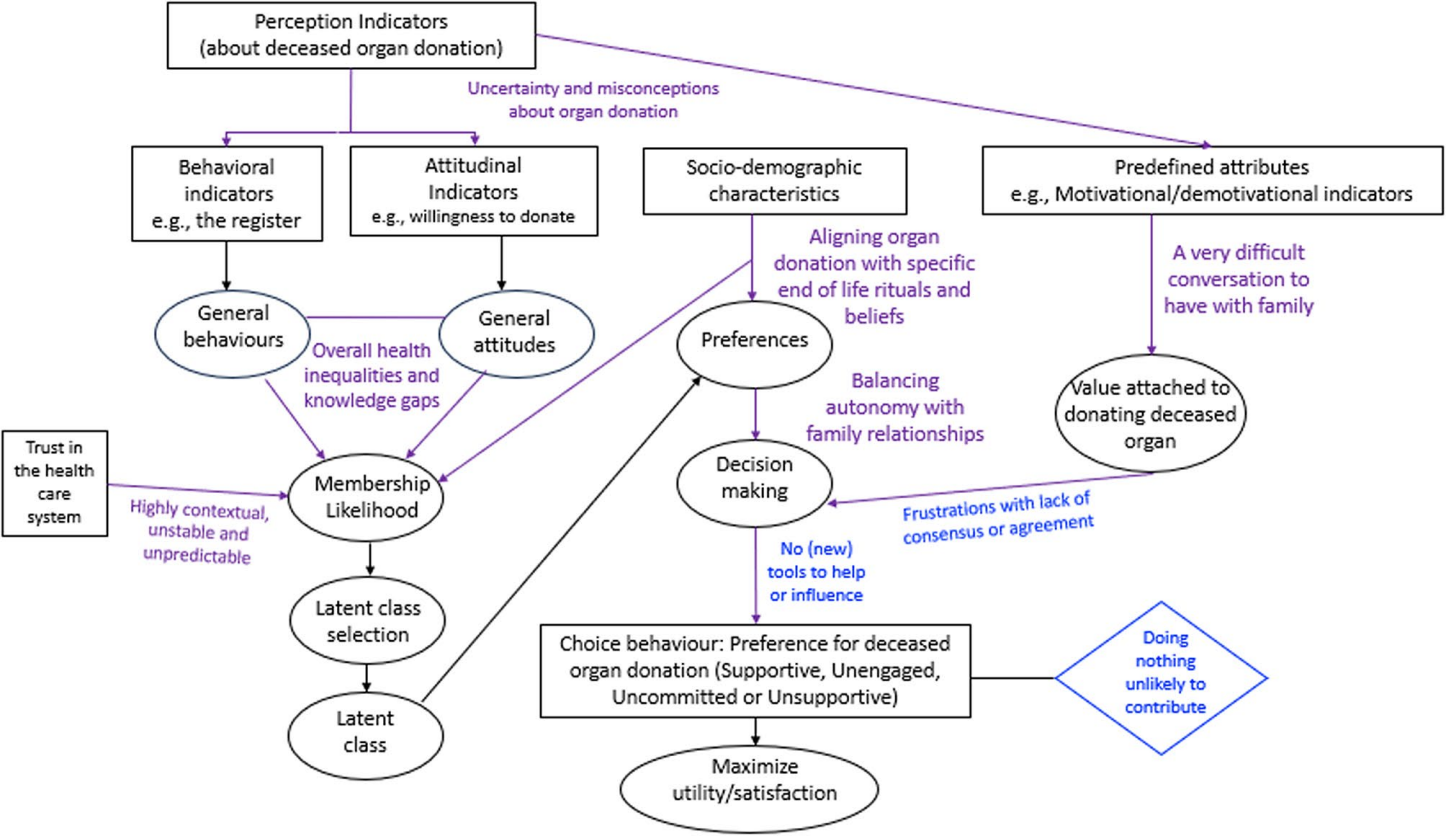
Integration of findings using the analytical and interpretive framework. Purple = qualitative interview data highlighting where additional ethnic minority perspectives overlap and potentially conflict with the intentions of the Act and where additional policy consideration may be needed. Blue = PPIE and interview data highlighting where additional ethnic minority perspectives overlap and potentially conflict with the intentions of the Act and where additional policy consideration may be needed. Black = quantitative data summary key results and/or signposted to in the manuscript and/or supplemental material. Factors in rectangles represent the variables that inform individual’s decision-making processes related to donating deceased organs, observable in the dataset, and those in ellipses are latent/unobservable variables and estimated from the model

Figure 1 presents a visualization of how the quantitative and qualitative findings were mapped against the analytical framework (rational choice theory). The integrated findings are presented in the discussion.

## Results

### Quantitative results

#### Public attitudes and reported behaviour towards deceased organ donation

**Fig. 2:**
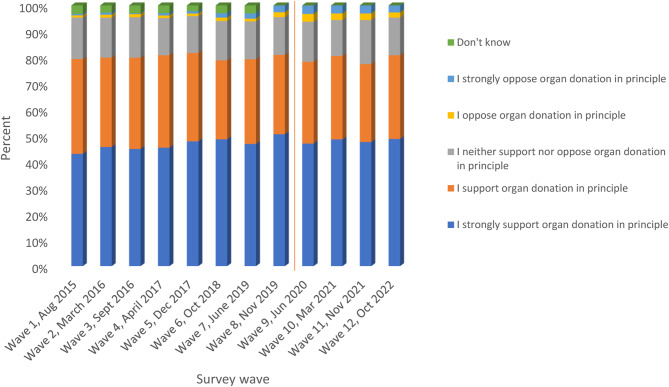
Public support for organ donation in principle; Source: NHSBT Organ Donation Attitudinal Tracker survey data (2015–2022). (Chi-squared statistic = 491.04, *p*< 0.001). Note: The red line demarcates results from survey waves before and after the law change

The results from the NHSBT Organ Donation Attitudinal Tracker Survey data showed that public support (those who reported being strongly supportive and/or supportive) for deceased organ donation in principle remained high and relatively stable over each wave, with around 80% of the population in England in support (Fig. 2). The Chi-squared test was statistically significant at the 1% level. This was similar before (wave 1 to wave 8) and after (wave 9 to wave 12) the law change, except in wave 11 (November 2021) where the proportion in support of organ donation was about 2% lower.

**Fig. 3:**
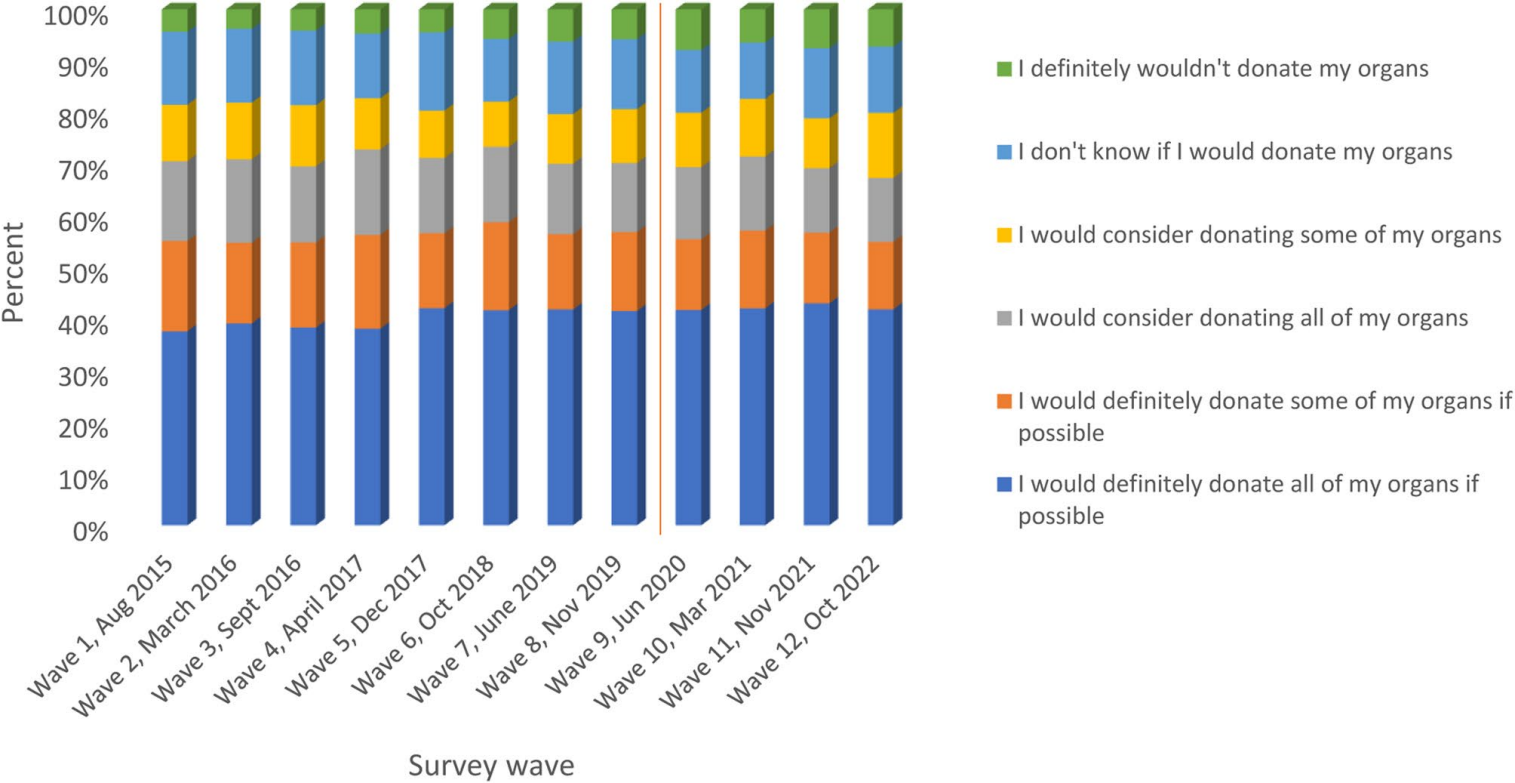
Willingness to donate deceased organs among the public; Source: NHSBT Organ Donation Attitudinal Tracker survey data (2015–2022) (Chi-squared statistic = 171.01, *p*< 0.001). Note: The red line demarcates results from survey waves before and after the law change

However, the proportion of the public that reported that they were willing to donate all or some of their organs after death was lower than those supporting organ donation as a general principle. On average, 56% of the population reported a willingness to donate all or some organs, 25% reported they would consider donating all or some organs, and the remaining proportions reported either that they were unsure or would not want to donate organs after death (19%) (Fig. 3).

**Fig. 4:**
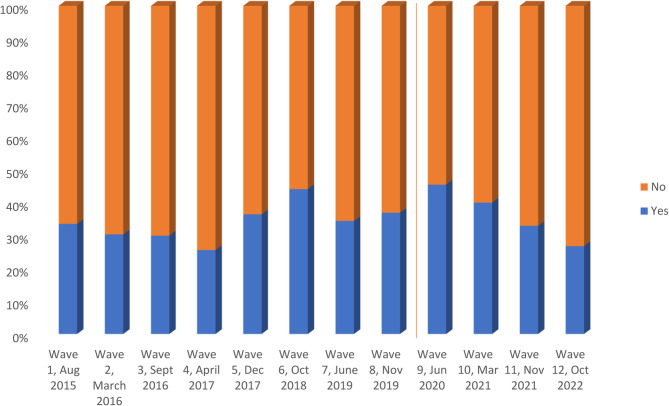
Public awareness of general organ donation publicity (those who had ever seen, read or heard a news item, advert, publicity, or other type of information on organ donation); Source: NHSBT Organ Donation Attitudinal Tracker survey data (2015–2022) (Chi-squared statistic = 326.23, *p*< 0.001). Note: The red line demarcates results from survey waves before and after the law change

There was relatively low public awareness of the general organ donation publicity (i.e., proportion who had seen, read or heard a news item), with 36% of the population aware. The lowest proportion of public awareness of organ donation publicity was reported in wave 4 (April 2017) at 26%; while the highest level of awareness was reported in wave 9 (June 2020) at 45%. The results show a decreasing trend in public awareness of organ donation publicity after the law change. The proportion of awareness declined by about 6% on average from 45% in wave 9 (June 2020) to 27% in wave 12 (October 2022) (Fig. 4). Overall awareness of the general organ donation publicity was 10% higher among the minority ethnic groups compared to the ethnically white groups. The top five sources of information were television (21%), articles in newspaper or magazine (10%), Facebook, Twitter, Instagram, or other social media platforms (9%), hospital, GP surgery or clinic (8%) and the radio (7%) (Supplementary file 5).

### Awareness of the law change

Results from analysis of additional surveys conducted to assess awareness of the new law and the changes to the organ donation system in England show that 58% of the public was aware of the law change (31% among minority ethnic groups) (Supplementary Table 3). The top five sources of information about the law change were Instagram (22%), online articles, news stories or adverts (16%), radio (16%), newspapers (15%) and television (14%) (Supplementary file 6).

### The NHSBT organ donor register

**Fig. 5:**
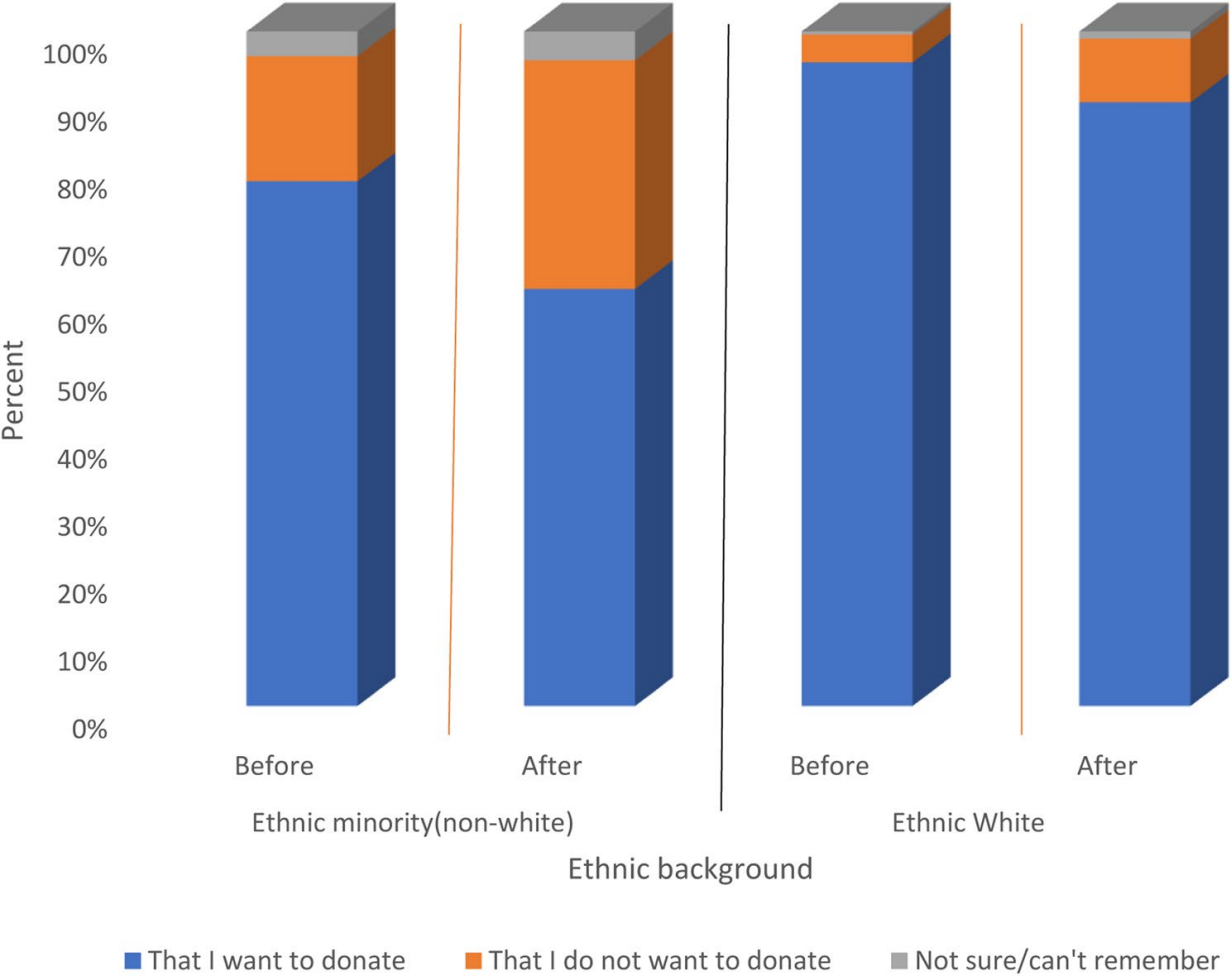
Reported decisions registered on the NHSBT Organ Donor Register. Note: results for before the law change were based on available data from wave 8 (November, 2019), and that of after the law change (June 2020 to October 2022) was based on average responses for four waves (waves 9–12). Source: NHSBT Organ Donation Attitudinal Tracker survey data (2015–2022) (Chi-squared statistic = 24.96, *p*< 0.001). Note: The red line demarcates results from survey waves before and after the law change

On average, 42% of the public had registered a decision on the NHSBT Organ Donor Register. Of those, 89% had registered a decision to donate and 10% had registered a decision not to donate. The remaining 1% who had registered a decision on the NHSBT Organ Donor Register could not remember the decision for which they had registered. Figure 5 shows the reported decisions registered on the NHSBT Organ Donor Register comparing white and non-white ethnic groups before (November 2019) and after the law change (June 2020 to October 2022). The results show a similar trend for both groups, except that, in all instances, the proportion registering to donate among those who self-described as ethnically white was higher compared to those in the non-white group.

For both groups the proportion registering a decision to donate fell after the law change, by about 16% in the ethnic minority group, and 5% in the ethnically white group. Also, the proportion registering a decision not to donate increased among both ethnic groups after the law change, an increase of about 15%, among the non-white minority group, and 5% among the white group.

### Talking about organ donation

**Fig. 6:**
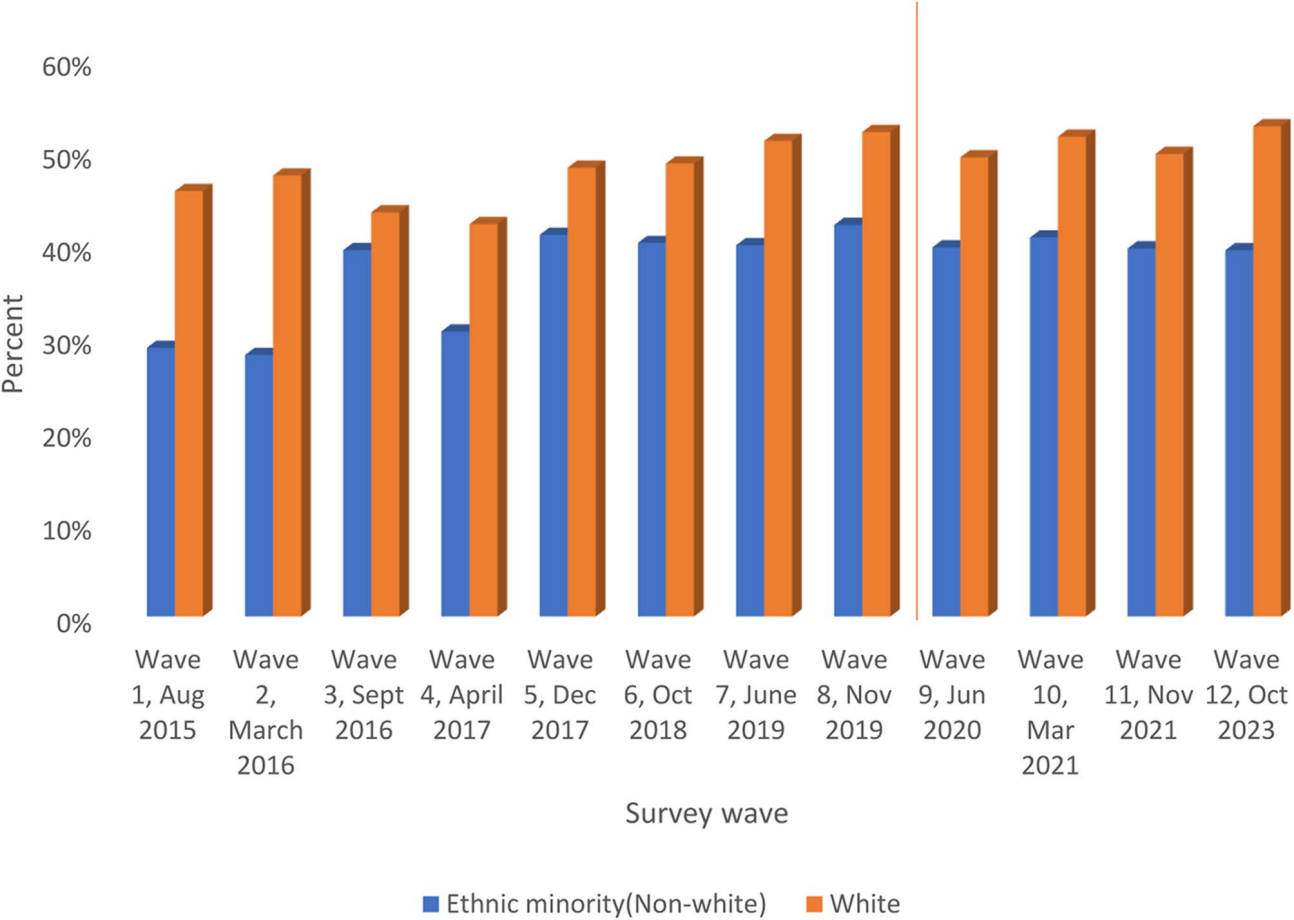
Proportion of individuals who reported having had a conversation with a close relation or family member by survey wave. Source: NHSBT Organ Donation Attitudinal Tracker survey data (2015–2022). (Chi-squared statistic = 77.52, *p*< 0.001). Note: The red line demarcates results from survey waves before and after the law change

The results in Fig. 6 show a rising trend in the proportion of the public reporting that they have had conversations about organ donation, but this did not appear to have been sustained in the period following the law change.

### Latent class (subgroup) analysis results

To identify subgroups of the population in terms of their views and potential behaviours, and to help identify which groups might benefit from interventions designed to encourage them to consider deceased organ donation more positively, subgroup analysis was undertaken. This subsection presents the results of the latent class (subgroup) model that was estimated subject to the level of motivation/demotivation (average motivation score) to donate deceased organs. Table 2 shows the results of the statistical tests used to identify the number of subgroups within the population with different inclinations towards deceased organ donation using survey wave 10 dataset, chosen for having the most diverse population subgroups (see Supplementary Table 4 in supplementary file for statistical test results for other survey waves). The corresponding number of latent classes/subgroups where the minimum of both the Akaike Information Criterion (AIC) and the Bayesian Information Criterion (BIC) was achieved was four.

**Table 2:** Test results to identify optimal number of population segments/subgroups, wave 10, March 2021

Number of classes/subgroups	Log-likelihood function	Number of parameters	Akaike Information Criterion (AIC)	Bayesian Information Criterion (BIC)
2	-3677.5	3	7361.00	7378.06
3	-3654.24	5	7318.47	7346.91
4	-3644.62	7	7303.23	7343.04
5	-3644.61	9	7307.23	7358.41
6	-3644.62	11	7311.23	7373.79

We therefore estimated a four latent class (subgroup) model. The regression results (Table 3) show latent class probabilities of 24%, 22%, 46% and 9%, respectively. These are the probabilities that a randomly chosen adult (16+) in England would belong to the first, second, third or fourth subgroup, respectively. The estimated latent class regression model has two main components. The first part of Table 3 presents the utility/motivation coefficients associated with deceased organ donation, and the second part shows the subgroup membership coefficients, capturing the impact of the characteristics on the probability of belonging to a particular subgroup. The membership coefficients for the fourth subgroups are normalized to zero to allow the remaining coefficients of the model to be identified in the estimation process [29].

The utility coefficients of motivation were all statistically significant at the 1% level for all the four subgroups of the population with similar preferences towards deceased organ donation. However, the motivation coefficients for subgroup 1 and subgroup 3 were positive and those of subgroup 2 and subgroup 4 were negative. This indicates that individuals in subgroup 1 and subgroup 3 were more positively motivated to donate deceased organs, while those in subgroup 2 and subgroup 4 were less positively motivated to donate deceased organ(s). Comparing the magnitude of the coefficients for the motivated subgroups, subgroup 1 placed more value on deceased organ donation (6.99[3.845,0.111]) than subgroup 3 (0.59 [0.383, 0.087]). Comparing the magnitude of the coefficients for the less motivated subgroups, individuals in subgroup 2 were less motivated to donate deceased organs (-1.03[-1.207, -0.930]) compared to those in subgroup 4 (-0.67[-0.858, -0.478]).

The regression results of the subgroup membership equation show that individuals in subgroup 1 were more likely to be older and female; less likely to be from North West England, North East England, Yorkshire and Humber, West Midlands, East Midlands and the South of England(excluding London); more likely to be white; less likely to be Christian or Muslim; much more aware of general organ donation publicity; more likely to support organ donation in principle; and more likely to be aware of the NHSBT Organ Donor Register, than those in subgroup 4 (Table 3).

**Table 3:** Four latent class (subgroup) estimates of preferences towards deceased organ donation

	Subgroup1: Supportive donors	Subgroup 2: Unengaged donors	Subgroup 3: Uncommitted donors	Subgroup 4: Unsupportive donors
	Coefficient [95% CI]	Coefficient [95% CI]	Coefficient [95% CI]	Coefficient [95% CI]
Share of population	23.8%	21.5%	45.6%	9.1%
**Utility function -motivation towards deceased organ donation**				
Motivation	6.99***[3.845,10.130]	-1.07***[-1.207,-0.930]	0.59***[0.383,0.799]	-0.67***[-0.858,-0.478]
**Class/subgroup membership function**				
Age	0.05[-0.011,0.111]	0.09**[0.007,0.170]	0.03[-0.035,0.087]	-
Sex (Female)	4.92**[0.831,9.004]	***19.90[15.165,24.638]	5.22**[1.132,9.312]	-
Region				
North West England	-9.42***[-14.656,-4.186]	-24.59[-29.631,-20.899]	-10.65***[-15.828,-5.468]	-
North East England	-3.62[-9.655,2.419]	-21.02***[-26.871,-15.159]	-5.36**[-11.374,0.663]	-
Yorkshire and the Humber	-8.72***[-14.234,-3.210]	-25.26***[-29.631,-20.899]	-10.25***[-15.719,-4.775]	-
West Midlands	-8.94***[-15.151,-2.719]	-9.99***[-16.178,-3.806]	-10.01***[-16.247,-3.777]	-
East Midlands	-11.38***[-16.722,-6.046]	-12.89***[-18.331,-7.454]	-12.29***[-17.561,-7.014]	-
East Anglia	0.05[-7.695,7.795]	-0.47[-8.282,7.347]	-0.35[-8.030,7.328]	-
South East England (excluding London)	-6.32**[-11.159,-1.485]	-7.13***[-12.202,-2.062]	-7.22***[-12.022,-2.426]	-
South West England	6.24[-2.489,14.975]	5.51***[-3.144,14.164]	5.07[-3.659,13.807]	-
Ethnic background (white)	10.51**[6.246,14.776]	9.59***[5.366,13.807]	9.97***[5.761,14.184]	-
Religion Christianity	-3.82***[-6.654,0.984]	-4.28**[-7.317,-1.251]	-3.29**[-6.127,-0.447]	-
Islam	-35.28[-29.631,-20.899]	6.68***[1.548,11.809]	3.37***[-0.746,7.490]	-
Organ donation (OD): OD publicity awareness	7.40***[2.954,11.853]	6.98***[2.394,11.575]	7.17***[2.749,11.585]	-
Support for OD	22.53[-155.309,200.362]	-11.67***[-19.219,-4.130]	7.25***[4.026,10.483]	-
Awareness of ODR	7.29***[4.264,10.316]	5.32***[2.095,8.545]	6.60***[3.548,9.645]	-
Constant	-26.23[-204.068,151.607]	-6.84***[-11.828,-1.845]	-7.66***[-12.263,-3.050]	-
Log likelihood	-3192.23			
Observations	2180			

Coefficient significant at 5% (*p* < 0.05) (**); 1%(*p* < 0.001)(***). The membership function coefficients for subgroup four are missing because they are the comparison subgroup. Figures in parenthesis are the 95% confidence intervals. OD represents organ donation. ODR represents NSHBT Organ Donor Register

In comparison to individuals in subgroup 4, those in subgroup 2 were more likely to be older, female, from all regions except South West England and London, and ethnically white. They were less likely to be Christians but more likely to be Muslims, more likely to be aware of organ donation publicity and the NHSBT Organ Donor Register, but less supportive of deceased organ donation (Table 3).

Comparing individuals in subgroup 3 to those in subgroup 4, those in subgroup 3 were more likely to be older, female, living in any regions other than South West England and London, ethnically white, less likely to be Christian but more likely to be Muslim, aware of organ donor publicity and the NHSBT Organ Donor Register, and supportive of organ donation in principle (Table 3).

### Characteristics of the four population subgroups with differing preferences towards deceased organ donation

**Fig. 7:**
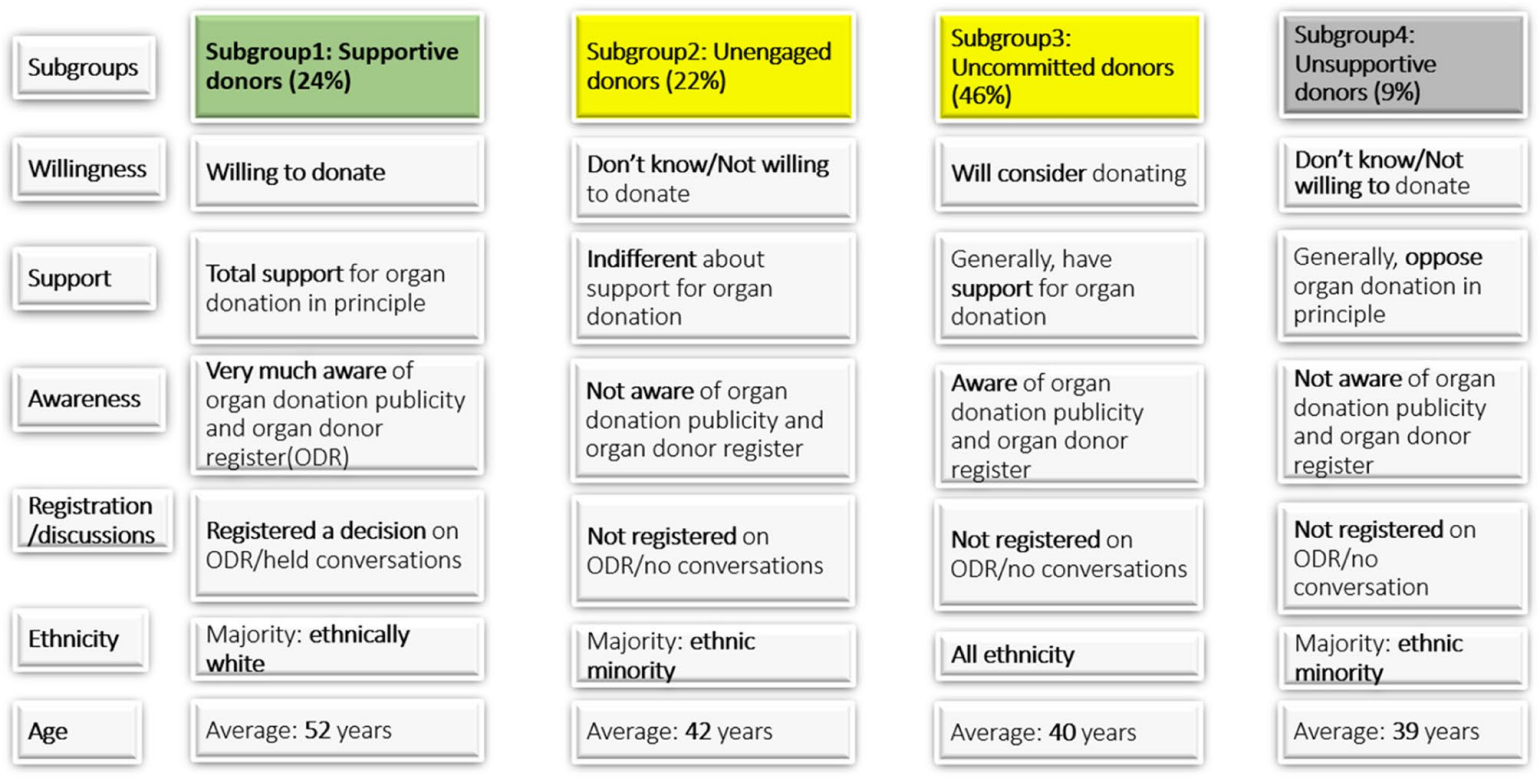
Summary of characteristics of individuals belonging to the four deceased donor subgroups (See Supplementary Table 5 in supplementary file for underlying statistics)

Further analysis of each of the identified subgroups showed that most of the individuals in subgroup 1 were willing to donate all or some organs when deceased, totally supported organ donation in principle, were highly aware of organ donation publicity and the NHSBT Organ Donor Register, had registered a decision on the NHSBT Organ Donor Register and had held conversations with close relations about their decision and intentions regarding deceased organ donation. Also, most of them were ethnically white. Their average age was 52 years. Based on these characteristics and the positive coefficient of motivation towards organ donation, we labeled this subgroup of the population as *“Supportive donors”.* This subgroup appears strongly to support deceased organ donation and is unlikely to be swayed in their views (Fig. 7).

Most of the individuals in subgroup 2 either do not know whether they will be willing to donate deceased organs or are not willing to donate; are indifferent about organ donation in terms of support in principle; and most were not aware of the organ donation publicity or the NHSBT Organ Donor Register. This subgroup was dominated by individuals from minority ethnic groups, with an average age of 42 years. We labeled this subgroup as *“Unengaged donors”* based on these characteristics and the negative coefficient of motivation towards deceased organ donation.

Generally, individuals in subgroup 3 reported that they would consider donating their organs after death, supported organ donation in principle, were aware of the publicity about organ donation and the NHSBT Organ Donor Register but most of them had neither registered a decision on the NHSBT Organ Donor Register nor had a conversation regarding their preference with close relations. This subgroup was made up of individuals from all ethnic backgrounds with an average age of 40 years. This subgroup was labeled as *“Uncommitted donors”* based on their characteristics and the positive coefficient of motivation towards deceased organ donation.

The fourth subgroup was labeled *“Unsupportive donors”* as individuals in this group generally do not know or are not willing to donate organs after death, as in the case of the unengaged donor group. However, individuals in this subgroup generally oppose organ donation in principle and are not aware of the organ donation publicity and the NHSBT Organ Donor Register. This subgroup was dominated by individuals from ethnic minority groups with an average age of 39 years.

### Public perspectives from interviews

We undertook interviews with 30 participants some of whom had played voluntary roles to promote deceased organ donation with the public. The majority were female (*n* = 19), of Black or Asian ethnicity (*n* = 24), Muslim (*n* = 18) and were either uncertain of their organ donation registered decision or had opted out (*n* = 24) (Further demographic details are reported in Supplementary Table 6). We report eight key themes which relate to potential issues or concerns which may be contrary to the intentions of the law change.

#### 1. Feeling it would be a (very) difficult conversation to have

For many individuals in ethnic minority groups, sharing their organ donation decision was perceived as a very difficult conversation to have with (some members of) their family. Even people whose personal rational choice was strongly in favour of organ donation were still reluctant to have a conversation with their immediate family and it was very common to delay or put off registering or talking about organ donation with family member(s).

*“It’s easier to go out into the world*,* but when you’re dealing with your own family*,* I’m acutely aware of how hard it is*,* I mean I was shocked by his [Son] response*,* he [son] was just dead against it*,* he [son] kept saying mum no*,* no*,* no*,* I want you to know I will be fighting it if anything happens to you. But what I couldn’t get was a definitive answer as to why*,* I couldn’t get past that initial anger and frustration. And I’ve not heard him talk so strongly really about anything pertaining to me. Now I’ve got this dilemma*,* I don’t want to hurt my family…but for the sake of not causing upset I’ve just kind of backed down. I may venture back to it*,* but I feel now is not the right time*,* he is expecting a child*,* so I’ve left it for now. ” (Female*,* Black*,* Christian (137))*.

#### 2. Balancing what individuals want with what their family expects

The autonomy and rational choice in life assumed to be realistic from the perspective of the law change (i.e., giving decisions to individuals rather than their families) did not necessarily easily translate to families where decisions are often shared or hierarchically made. Individuals who were not necessarily seen as final decision makers in many situations (e.g., younger people, women, second or third siblings, etc.) frequently encountered barriers when trying to share their personal choice or make their organ donation decision known. For some people, the rational choice was not theirs to make, and the choice belonged to their family or wider community.

*“I registered*,* I was so happy and then I got a message – “now it’s time to tell your family” – I thought really!? Why*,* why are you making me do this – is this not enough? Especially for us in an African setting women are a bit submissive to their husbands and so for every decision that you take it’s got to be like both making this decision” (Female*,* Black*,* Christian*,* 138)*.

*“It doesn’t matter what I do*,* I can register or not*,* but I know if the time came my husband wouldn’t allow it – it is him that needs convincing not me.” (Female*,* Ethnically white*,* Muslim*,* 242)*.

*“It is probably something we need to talk about*,* it has to be a family environment because I’ve got 4 siblings there are 5 of us and from the South Asian Tradition it is the eldest sibling that will carry the burden and make decisions. So*,* if my other brother knows exactly what mum and dad want*,* their wishes will be carried through*,* but it is a very intimate conversation.” (Male*,* Asian*,* Sikh*,* 156)*.

#### 3. Feeling unsure and ill-informed about organ donation

People involved in paid or unpaid roles to help promote organ donation highlighted the importance of individuals sharing their choices by making them known through the NHSBT Organ Donor Register and/or encouraging them to talk about their organ donation decision with relatives, but many felt ill-equipped to answer more detailed questions about organ donation such as how, when, and where deceased organ donation comes about. This additional information is often needed by people in order to make a rational choice concerning organ donation. People who were much more reluctant to donate their organs remained so and many people tasked with promoting organ donation after the law change still felt they had insufficient knowledge, access, and communication skills to reach those harder to engage groups and individuals.

*“The other thing is the question of how this is done*,* I had a guy ask me*,* “now if I want to donate my heart won’t they kill me faster because they want to have my heart ticking” So what is it I’m donating*,* at what point in time*,* when would it happen*,* we need to do so much more work to sensitise the whole process – people don’t understand just how much we don’t know*,* we are just getting our heads around blood for god’s sake and now you want us to do this!” (Female*,* Black*,* Christian*,* 138)*.

There were frequently deep-seated cultural attitudes which influenced views and perceptions of deceased organ donation, often related to associations with trafficking and selling organs and dismembering of bodies. These lay perceptions and views (particularly from people from ethnic minority group) were perceived as rational by individuals, and they negatively influenced their choice and decision to donate their organs.

*“It’s*,* dark*,* for us it is to do with witchcraft*,* with sacrifice*,* people go out get money so they can donate to their witchdoctors. I even remember growing up my mother would say if I don’t come home immediately somebody’s gonna cut your ear off*,* somebody will take your eyes. So*,* in Africa this is what it is witchdoctors who need eyes and breasts! (Female*,* Black*,* Christian*,* 138)*

#### 4. Wanting to refocus on the high need for transplants amongst minority ethnic groups

Most people from ethnic minorities felt that messaging related to deceased organ donation needed to increase the focus on the unmet need for transplants, especially in relation to needs of people from minority ethnic backgrounds. People wanted the messages to include the high costs of dialysis compared with transplants, the waiting list for organs and the consequences when people are unable to get an organ. Many people from these minority ethnic groups felt that people would be able to more easily make a choice to donate their organs if they knew that more people within their community needed transplants and that they would benefit and have a better quality of life if they received a transplant.

*“I mean it is our people that are dying*,* I didn’t know that*,* and that is the message that needs to be out there” (Female*,* Christian*,* Black*,* 137)*.

*“A friend of mine is on dialysis*,* refusing an organ*,* I said to him do you know how much you are costing me!? I think if more people knew the real scale of the problem*,* they would help*,* there are few people in the world who don’t want to help*,* very few” (Male*,* Asian*,* Hindu*,* 149)*.

*“I’ve asked so many questions to people and they’ve all said*,* “It’s not affecting us*,* so why should we bother?’” (Female*,* Asian Muslim*,* 242)*.

#### 5. Lacking in trust and the need to build it

Misunderstandings, misinformation, and fake news (that all seemed rational to individuals, but which negatively influenced their organ donation choice) were very commonly discussed, often fuelled by historical mistrust of state agencies (including the NHS) among people from minority ethnic groups and certain faith groups, especially Muslim or Jewish people. Some people thought wrongly that the law had been modified to include families in decisions, following protests from organisations representing faith groups. The COVID-19 pandemic, including controversies about vaccines, and the murder of George Floyd were frequently cited by interviewees from a minority ethnic background as potential barriers and explanations as to why people might have opted out in masse in certain communities soon after the law change. Many people, particularly from a Black or Asian ethnic background or a Muslim faith, who had opted out had done it in response to a text message or word of mouth which contained inaccurate information relating to a deadline to opt out, after which, it was claimed, their organs would become the property of the UK Government.

*“In our minds the NHS is government*,* too much has happened historically where the NHS has taken bodies*,* they’ve done so much that everybody is so nervous*,* so you come out and say*,* ‘we’re going to take your organs we are like no you are fucking not - everyone get your name off’ and that’s essentially what happened” (Female*,* Black*,* Christian (139))*.

The majority of those from minority ethnic groups felt that a trusted community leader was a key voice in helping to bring a rational debate about organ donation into their communities. NHSBT had recently set up several schemes to support this grass roots work – but many were struggling to identify the impact of these schemes and felt that the performance measures used by NHSBT (e.g., number of people registering on the NHSBT Organ Donor Register, number of people at events, etc.) were too blunt and missed the fact that most people had never before heard of organ donation and would need multiple engagements to enable them to make a rational and informed choice based on correct information.

#### 6. Bringing organ donation, and end of life care, rituals and beliefs closer together

Although most people (irrespective of ethnicity or faith) felt the law change was a good idea in principle, many were not confident that their cultural preferences and rituals related to death, repatriation of the body and burial were consistent with the legislation. There was also misinformation regarding the care taken to retrieve organs and the physical appearance of the body afterwards. For these people, this had a major impact on their likelihood of deciding in favour of deceased organ donation. This was another example whereby support for the law in principle was counteracted by other public knowledge and understanding (sometimes incorrect) that swayed the choice towards not donating organs.

*“The law is right*,* but I’ll tell you*,* imagine my body arriving home*,* you know it has a scar or whatever. People check your body left*,* right and centre*,* it is not like here where you are all wrapped up*,* they will wash you*,* smear you in Vaseline – I mean the whole village. So*,* imagine my illiterate mother seeing her daughter’s body in bits and pieces. She will scream – she will not allow me to be buried before she has the answers. You get the point? I was talking with a Nigerian man on Sunday he said*,* ‘If I arrived home and I do not have some body parts they are not going to put me in the main cemetery*,* because I’m not full” (female*,* Black*,* Christian*,* 139)*.

Others, particularly people from the Jewish faith, were concerned over the definition of death (irrespective of the law change) and had opted out in protest that the law did not go far enough to provide clarity and reassurance that individual faith perspectives would be protected, including their views on brain death.

*“I’ve opted out*,* the nebular statement saying religious concerns will be noted is not good enough given the weight and seriousness with which Judaism views end of life issues. This is nothing to do with organ donation – Judaism supports that – it is brain death. Now medicine is moving at the speed of light*,* this might not be an issue in 5–6 years. But there is no black and white*,* every case is on its merits*,* that is why the nurses need better training*,* but the numbers are so small [of people eligible and go onto become organ donors]*,* the guilt I felt for opting out was relatively low [because I’m so unlikely to become a deceased organ donor]” (Male*,* White*,* Jewish (192))*.

#### 7. Lack of consensus among faith and community leaders on organ donation

Most people from a Muslim faith felt frustrated by the lack of clear and consistent messages from their religious and/or community leaders. They reported that such people either could not agree or were reluctant to engage with organ donation. Many people felt that the reluctance to discuss and come to a consensus on organ donation was a matter of power and control rather than anything directly related to whether organ donation is deemed permissible or not in Islam. The resultant uncertainly seemed to sway people towards a choice not to donate.

*“Where I live*,* we got a lot of mosques*,* a lot of mosques*,* and it is like if I am running an event supporting this or that*,* then guaranteed the guy across the road is running an anti-event*,* I mean why can’t they just get on and get on with it. I’ve been campaigning for a long time and honestly it is so tired now*,* fuck em*,* this is about nothing more than power and control – and it’s so frustrating to listen over and over to the endless bickering and same old rants about what is in the book or not*,* permissible*,* or not. I mean it wasn’t written for this [organ donation] end of story! (Male*,* Asian*,* Muslim (165))*

*“There is her [Imam]*,* but no he [Imam] is against it. But I mean we all do things that are not in the book*,* we all eat Nandos and this and that…we just don’t know. But I do know Islam is a very very giving religion*,* it really is. I guarantee if they just came out and said it was permissible*,* we would all do it” (female*,* Asian*,* Muslim (242))*.

*“Yes*,* they just say no its not allowed…But the thing is you can give a kidney whilst you are alive*,* so how does that work*,* because you are not going back in your grave complete*,* do you understand what I am saying?” (Female*,* Asian*,* Muslim (242))*.

#### 8. Doing nothing to share an organ donation decision causes (more) problem

The legislation provides several options for people to make a choice. Some people were very happy with the idea of deemed consent (i.e., the choice to do nothing in life and thus be presumed to have no objection to being an organ donor) as it gave them one less thing to do in an otherwise very busy life. Others felt that organ donation was very important and felt guilty that they had not thought about it or done more to convey their decision by registering or talking about it to their families. People who did not know about the law change (and who supported organ donation) sometimes felt embarrassed or naive that they did not know and subsequently worried about what they should or needed to do next to convey their decision. Most people still felt that if somebody did not register or discuss organ donation in life, and so came under deemed consent, the family would not feel sufficiently reassured that this was a legitimate and rational choice. The law did not provide any (new) ways to alleviate any of the concerns (discussed above) from minority ethnic perspectives.

## Discussion

Changing the law has had little impact on the general public’s overall, in principle, support for organ donation which has remained high and stable (80+%). Further, it does not appear to have influenced people’s willingness to become deceased organ donors which is lower at 56% with considerable variation in what people wish to donate.

Ethnic minority support and willingness to donate remains lower (20+%) than in the white population. At the same time, we also found that individuals from minority ethic groups could potentially be supportive of organ donation, but family, and cultural factors sometimes tended to prevent them from doing so. Thus, it was not always the individual’s decision to make, contrary to the assumptions underpinning the law. There were also (very) low levels of understanding of deceased organ donation and how it comes about in ethnic minorities as well as concerns about whether the processes of organ retrieval aligned with their cultural beliefs and preferences. This knowledge and experience can contribute to a decision that is perceived to be perfectly rational from the individual’s perspective. Their choice is, however, often perceived as irrational and misinformed by professionals and at odds with the principle underlying the law, which assumes that people will make a personal rational choice based on public information campaigns and official sources of information [36]. The law also assumes that the default of ‘opted-in’ will be influential whereas for a lot of people there are plenty of factors pushing them in the opposite direction thereby blunting the impact of the principle that everyone is a potential organ donor.

The level of awareness of general organ donation publicity was relatively low (36%) and unstable over the surveys but awareness of the law change was, perhaps surprisingly, 58% in the white population but lower in ethnic minority populations (31%). Additionally, minority ethnic groups were often unaware and shocked by the long waiting time for organs, and frequently wanted awareness of the adverse impacts of the lack of organs on their communities to be increased. This information was needed to inform their rational choice to donate their organs. The lack of information and level of misinformation were exacerbated by frustrations with inconsistencies and lack of consensus on organ donation on the part of people in positions of leadership, whom many felt should take a more positive role in addressing these inequalities. People in leadership positions can control the narrative and knowledge which community members use to make their decisions about organ donation.

The number of people registering on the NHSBT Organ Donor Register has stagnated. Of those registered, 89% have opted in and are predominately white; about 11% have opted out and are predominately non-white. However, these findings relate to the early period of the implementation of the new law which was marked by a series of extraordinary events including COVID-19, the murder of George Floyd in the US and vaccine hesitancy which contributed to a narrative of government conspiracies directed at harming members of ethnic minority groups, including, by implication, the NHS, and resultant mistrust. We also observed the consequences of misleading targeted campaigns against organ donation which rapidly and easily spread due to social media. Minority ethnic families frequently used WhatsApp to talk to their relatives overseas as well as to access community information. Messages circulated within WhatsApp groups tended to encourage people to opt out of organ donation, which was again a rational choice for these individuals when faced with believable misinformation in an atmosphere of mistrust.

There was an overall increase in the proportion of the public that had had conversations about organ donation. However, the intentions of the law change (to give decisions to individuals) were frequently misunderstood, and arguably difficult to be easily translated into families where decisions of any kinds are often arrived at collectively, not just those related to organ donation.

Of the four identified population subgroups, supportive donors and unsupportive donors are unlikely to respond (positively or negatively) to interventions designed to raise the consent rate. Unengaged donors displayed the most uncertainty about organ donation and may respond to targeted interventions to promote and raise awareness of organ donation. Apart from the supportive donors, most of the individuals in the other three groups had not discussed their organ donation views or preferences and may benefit from more opportunities to talk or register on the NHSBT Organ Donor Register (especially uncommitted donors). This is important as, although most people supported the changes, the presumption of consent left gaps in people’s knowledge in that it left them wondering what they needed to do while alive, what would happen if they or their relative who died was eligible for organ donation and critically what they would do if they did not know what their relative who died had wanted. Thus, the assumption underpinning the 2019 Act – making it easier for individuals to make their organ donation decisions, and that these decisions would be informed by factually correct information is far from an accurate description of the situation of many people, especially in some ethnic minority families. Some people are making decisions based on lack of knowledge or misinformation, in a context of mistrust, feel no more supported or empowered to make their decision or reassured that it would be upheld in the event of their death. The law has not helped mitigate the lack of consensus in some faith communities and in some ways may have made things worse by encouraging the spread of misinformation. People in roles designed to educate and encourage organ donation in ethnic and faith communities are struggling to clarify and explain the law in ways that reassure and bring about the intended behaviour change.

### Strengths and limitations

A strength of this research is the theory-informed mixed-methods design (population surveys, latent class analysis and semi-structured, in-depth interviews) with a particular emphasis on groups traditionally underrepresented in research in general and specifically in organ donation. This enabled not just a description of trends but also integration of additional causal explanations and contextual features to help identify the practical policy implications. Our theoretical framework helped in exploring highly complex decision making and the strengths of the mixed-method design were shown in the additional issues uncovered from interviews with ethnic minorities in the context of analysis of representative population survey data on attitudes and behaviours towards deceased organ donation.

The findings also reveal some of the limitations of rational choice theory, namely, its focus on individual decision-making whereas for many respondents, organ donation decisions involve more than one person in a family context. According to the intention of the 2019 Act, the potential donor makes a choice during life and then when they die their family members are supposed to honour their relative’s rational choice. In practice, in some families, other family members make these organ donation decisions on their behalf. Rational choice theory also does not explicitly take into account that the potential organ donor likely died in tragic circumstances and family members’ behaviors will be emotionally (not purely rationally) based, and, in the highly emotional crisis context, the decision will be influenced by personal biases, intuitive reasoning and a fight or flight survival instinct. Despite the ambitions of the law change some people still had to go along with the choices of the community or family no matter how ill-informed it was or whether it matched their own preferences. The role of the family is reported elsewhere [37]. For decisions that must be weighed up and made quickly in tragic circumstances (such as in organ donation), rational choice theory thus only partly explains the intended behaviour change assumed in the legislation. On the other hand, this study was able to contribute to understanding the role of public knowledge and related logic, especially among some ethnic minority groups, in making what was from their perspective a rational choice.

Our study is novel in that studies in this field have tended to look at overall trends without addressing sub-population nuances and therefore have been unable to highlight new or more targeted interventions to address (increasing) inequalities in organ donation.

The main limitation of this study is that the authors were not involved in the survey questionnaire design or data collection and so were limited in the latent class modelling by the available variables. Overall, the model predicted 84% of the factors associated with belonging to a given organ donor subgroup. Future studies should help to account for the remaining 16% of the factors not accounted for in this study. Also, with more variables available, it might have been possible to categorise individuals in the sample more completely rather than being limited to a blunt ‘white’ or ‘non-white/ethnic minority’ category. Further, the surveys were repeated cross-sections, not longitudinal, so we were unable to explain changes over time, including the sequence of events which may have influenced public attitudes to deceased organ donation, as well as the possibility that individuals might transition from one subgroup to another over time.

### Implications of the study for policy, practice and research

To date, the law change in England from opt-in to ‘soft’ opt-out appears to have had little impact on factors known to influence consent rates or in addressing inequalities in organ donation. Unsupportive donors and especially those from minority ethnic communities are unlikely to be swayed by generic mass media campaigns. Agencies tasked with promoting organ donation may benefit from targeting uncommitted donors to encourage them to express their organ donation decision, and unengaged donors to address their exposure to likely mis/information, as well as community confusion and uncertainty. Interventions need to take account of public knowledge and perceptions that are very difficult to challenge or change and the (significant) role of the family as well as wider community influencers on attitudes, preferences and decisions. More and new opportunities need to be created for people to register and/or update their organ donation decisions over time. Future surveys to monitor public attitudes towards organ donation could be longitudinal in nature to enable the analysis of both time-invariant factors, and those factors that change over time so as to fully unpack the issues that affect public decisions towards deceased organ donation.

## Conclusion

Despite a high apparent level of support for the principle of organ donation, individuals are far from unanimous when it comes to their personal willingness to donate their organs after death. If consent rates to deceased organ donation are to be raised in England in future, attention needs to be given to engaging with subgroups who are sceptical, uncommitted or who have thought little about donation, especially those from ethnic minority groups.

## Electronic supplementary material

Below is the link to the electronic supplementary material.

Supplementary Material 1.

## Data Availability

The data analysed in this study are subject to licenses/restrictions. The authors do not have the permission to publish the dataset. Requests to access these datasets should be directed to https://www.nhsbt.nhs.uk.
