# Supplementary material for: Subgroup differences in public attitudes, preferences and self-reported behaviour related to deceased organ donation before and after the introduction of the ‘soft’ opt-out consent system in England: mixed-methods study
Source: BMC Health Serv Res. 2024 Nov 21;24:1447. doi: 10.1186/s12913-024-11821-3 (PMC11580530; doi:10.1186/s12913-024-11821-3)
Supplement: Supplementary file 1 — Supplementary Material 1. [file 12913_2024_11821_MOESM1_ESM.docx]

**Supplementary files**

**Supplementary file 1: An overview of the implementation of the soft opt-out into the previous opt-in system in England**


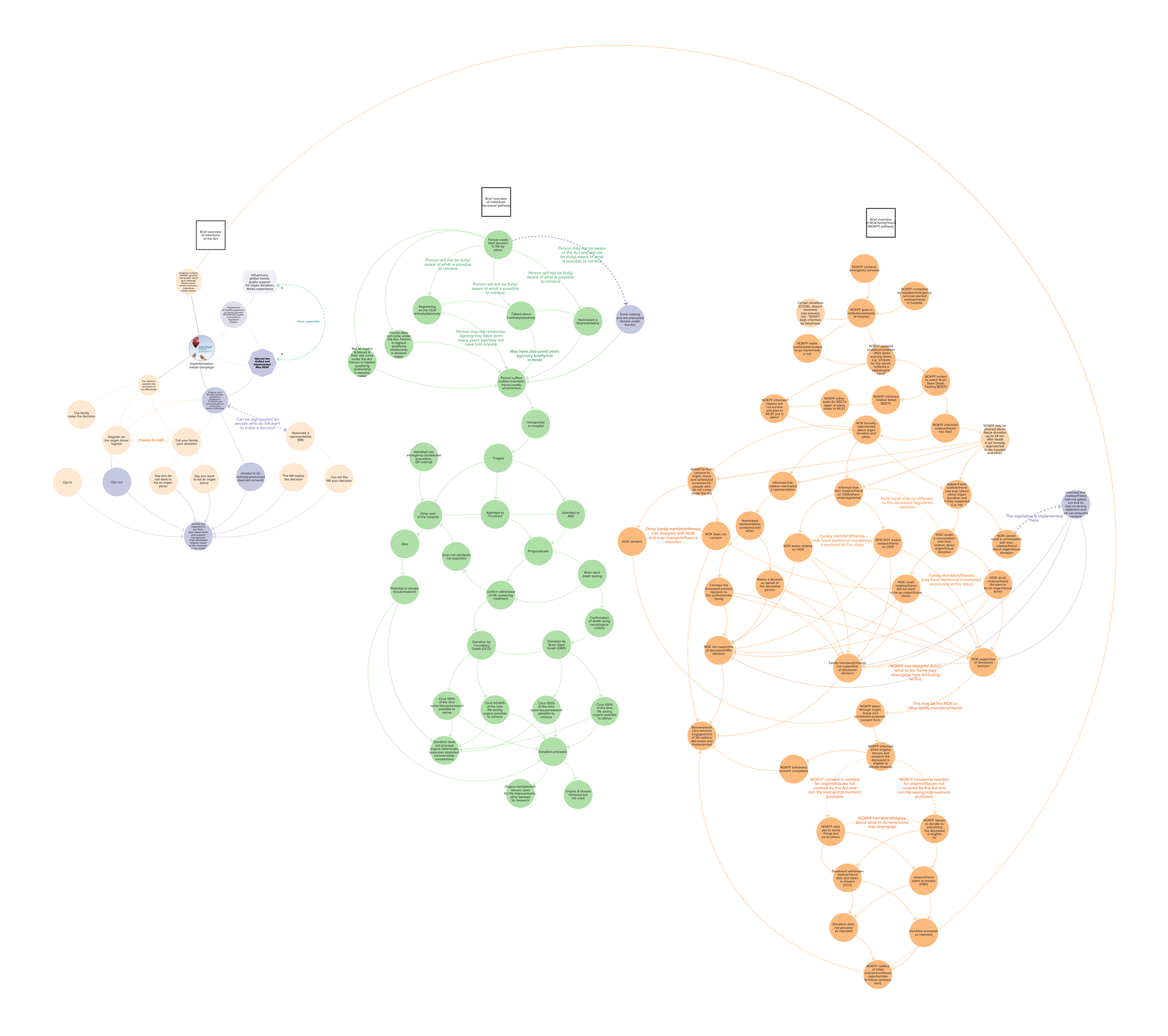


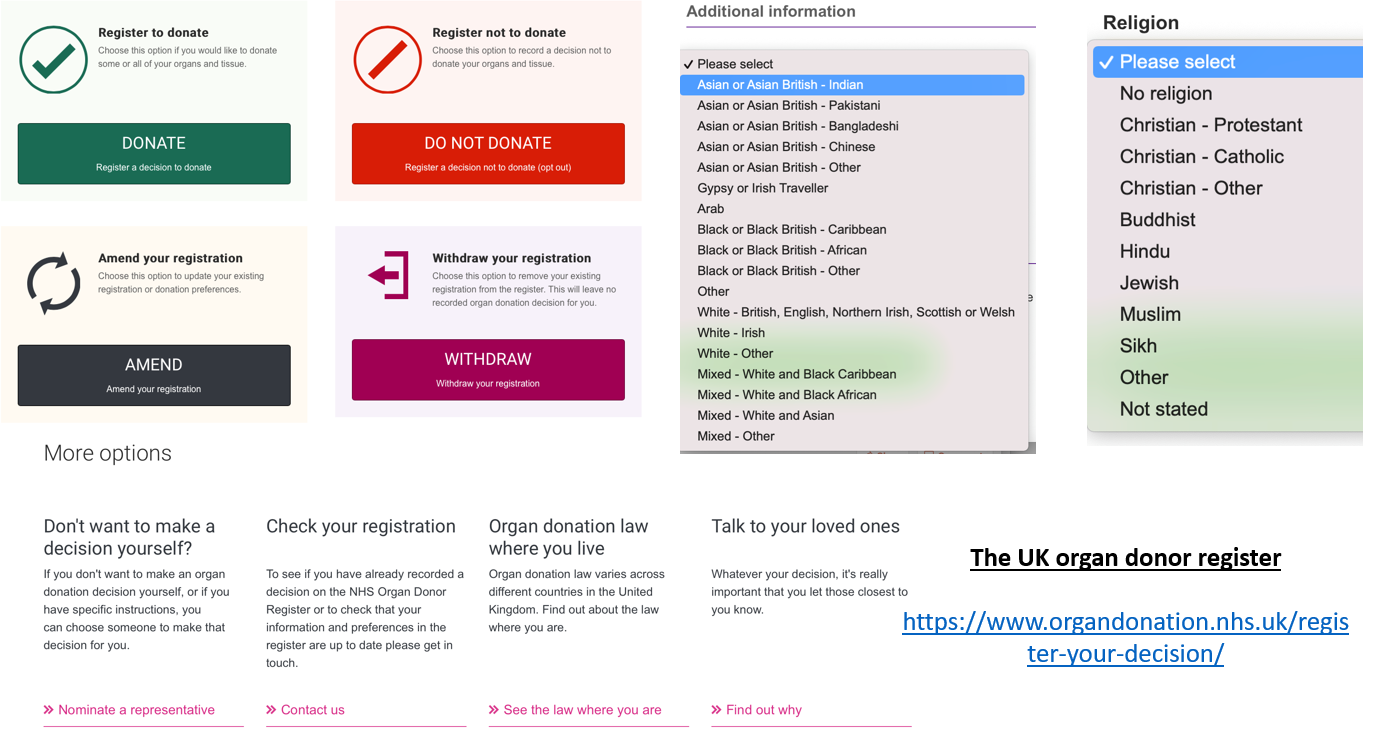


**Supplementary file 2: Interface of NHSBT Organ Donor Register**


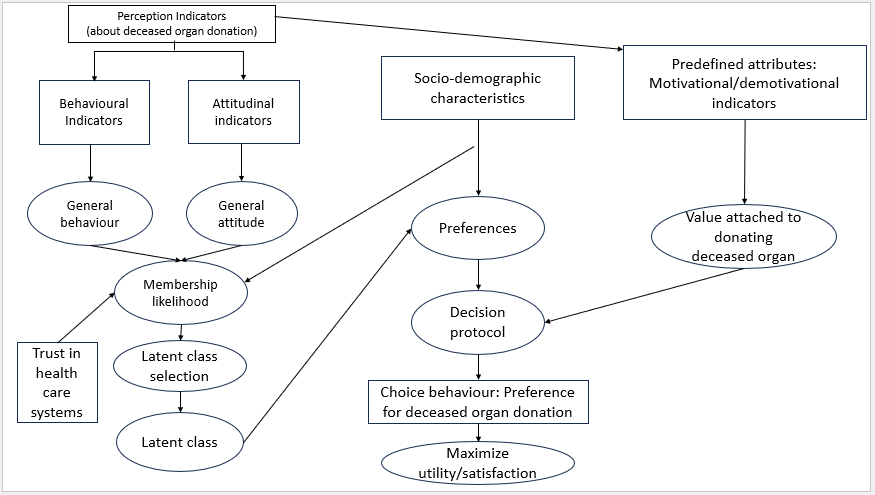


**Supplementary file 3: Analytical and interpretive framework for preferences towards deceased organ donation, latent class membership and population subgroups**

Factors in rectangles represent the variables that inform individual’s decision-making process about donating deceased organs that are observed by researchers, and those in ellipses are latent/unobservable by researchers. All these factors affect the value/satisfaction an individual attaches to becoming a deceased organ donor. General perceptions about deceased organ donation affect the general behaviour, attitude and the probability of individuals belonging to a specific deceased organ donor subgroup. The heterogeneous deceased donor subgroups are assumed to be formed, among others, by individual differing perceptions, behaviour, and attitudes towards deceased organ donation. The socio-demographic characteristics of individuals such as age, (health) educational level, ethnicity, religion, and life experiences are assumed to also affect the probability of an individual belonging to a given deceased donor subgroup.

The membership likelihood function provides the foundation for the formation of heterogeneous deceased donor subgroups. Though unobserved and statistically determined, it indicates the probability of individuals belonging to a given deceased donor subgroup.

**Supplementary Table 1: Sample distribution of respondents of the attitudinal tracker survey data per** **wave**

| **Wave** | **Excluded sample** | **Sample used** |
| --- | --- | --- |
| Wave1 | 12 | 1,488 |
| Wave2 | 13 | 1,484 |
| Wave3 | 4 | 997 |
| Wave4 | 10 | 1,489 |
| Wave5 | 10 | 1,590 |
| Wave6 | 37 | 1,821 |
| Wave7 | 35 | 1,767 |
| Wave8 | 21 | 1,775 |
| Wave9 | 17 | 1,781 |
| Wave10 | 29 | 2037 |
| Wave11 | 23 | 2,145 |
| Wave12 | 43 | 2,151 |

**Source: NHSBT Organ Donation Attitudinal Tracker survey data (2015-2022)**

**Supplementary Table 2: Sample Distribution of respondents of the Law Change data per survey wave**

| **Survey wave** | **Sample** |
| --- | --- |
| Wave1 | 1,261 |
| Wave2 | 1,262 |
| Wave3 | 1,278 |
| Wave4 | 1,277 |
| Wave5 | 1,299 |
| Wave6 | 1,280 |
| Wave7 | 1,231 |
| Wave8 | 1,274 |
| Wave9 | 1,278 |
| Wave10 | 1,275 |
| Wave11 | 1,005 |
| Wave12 | 1,270 |
| Wave13 | 1,233 |
| Wave14 | 1,277 |
| Wave15 | 1,282 |
| Wave16 | 1,229 |
| Wave17 | 2,502 |
| Wave18 | 1,273 |
| Wave19 | 1,282 |
| Wave20 | 1,277 |
| Wave21 | 1,280 |
| Wave22 | 1,275 |
| Wave23 | 1,274 |
| Wave24 | 1,280 |
| Wave25 | 1,269 |
| Wave26 | 1,276 |
| Wave27 | 1,272 |
| Wave28 | 1,275 |
| Wave29 | 1,270 |
| Wave30 | 2,551 |
| Wave31 | 2,513 |
| Wave32 | 2,556 |

**Source: NHSBT complementary survey to monitor the awareness of the law change (35 waves (2018-2022)**

**Supplementary file 4: FORM I. Topic Guide/ public interviews/ IRAS no.  297313 /Version 1/ 08.02.21**

**Evaluation of the Organ Donation (Deemed Consent) Act, 2019**

**Organ Donation Conversations – Member of the public interview topic guide**

Interviewees to include the general public including people who: support organ donation, do not support organ donation, support the changes in law, do not support the changes in law, people not associated with any organisations who promote organ donation as routine practice (e.g., health charities) people from BAME, minority and underrepresented groups and faith groups.

**Introductions**

- Researcher and overview of project
- Check time available for interview
- Consent
- Ensure participant knows we are there to listen to them and their views, there are no right or wrong answers or judgements.

**General views on Organ Donation**

- Unpack personal views on organ donation (sample questions)
- Where did your views come from?
- Have you changed your views on organ donation?
- Have you any prior experiences of organ donation?
- Are you registered on the ODR?

**Views and behaviours on the changes to consent for organ donation**

- Unpack views on the changes to consent to organ donation and any changes in behaviour (sample questions)
- How do you feel about the changes to consent for organ donation?
- Why do you think they changed the law?
- When and where did you first hear about them?
- Can you explain to me what the changes are?
- What difference would you like to see the changes make?
- What did you do when you heard about the changes e.g., register on the ODR talk about it, find out more etc? If registered on the ODR find out if they indicated their religion. If had a conversion unpack how this conversation went, when and with whom etc.
- Did anybody else in your family, friends, colleagues do anything?
- Anything else you would like to say about the changes to consent for organ donation?

**Media Campaign**

- Unpack influence of the media campaign, understanding of key messages and any recommendations for future campaigns (sample questions). *Show interviewee samples of the implementation media campaign.* Are you specifically aware of the BAME campaign? (Please give examples).
- Do you remember seeing any of these materials?
- What do you think about it?
- What did you do when you first saw it?
- What recommendations would you make for future campaigns?
- *‘Engaging with BAME and other minority and underrepresented groups Is a priority for the various organisations involved in organ donation to make it easier for people to make their organ donation decision while they are alive, and therefore easier for the family after we die as they know what we wanted to happen’* what would you recommend to better engage with these groups to achieve this?
- *The Opt-Out figure is not high (around 3%) yet around 80% of these are from BAME and faith backgrounds we do not understand why that is happening.* What do think about this, does it surprise you, why do you think this is the case?

**Impact of COVID**

- ‘The changes in law were passed in 2019. Government and various organisations involved in organ donation were part way through an advertising campaign when the COVID 19 pandemic started. The remainder of the planned advertising was cancelled.’ Unpack interviewees views on any influences of COVID 19 on implementation of the change in law and in the future. (sample questions)

Do you think the COVID 19 pandemic influenced the implementation of the changes in law?

- Do you think the COIVD 19 pandemic has influenced peoples’ views and behaviours on organ donation?
- Do you think the COVID 19 pandemic will have any influence on the numbers of people who consent to organ donation in the future?

*The BAME population has been disproportionally affected by COVID.*

Do you think this will (or has had) any impacts on the attitude of BAME population to organ donation. (unpack positive and negative impacts)

**Sample probes and prompts**

You mentioned…can you tell me more about…?

That’s interesting, can you tell me more about…?

Why do you think that is?

How has that been for you?

What do you think about….?

Why/why not?

Can you tell me a bit more about that?

Anything else you would like to say about that?

**Supplementary file 5: Sources of information about general organ donation publicity; Source: NHSBT Organ Donation Attitudinal Tracker survey data (2015-2022)**

**Supplementary Table 3: Public awareness of the law change**

| **Wave** | **Yes** | **No** | **Don’t know** |
| --- | --- | --- | --- |
| 1 | 52.8 | 39.1 | 8.1 |
| 2 | 55.3 | 37.5 | 7.1 |
| 3 | 36.3 | 55.1 | 8.6 |
| 4 | 38.4 | 53.4 | 8.2 |
| 5 | 58.4 | 35.7 | 5.9 |
| 6 | 59.2 | 34.2 | 6.6 |
| 7 | 51.1 | 40.8 | 8.1 |
| 8 | 55.7 | 37.1 | 7.2 |
| 9 | 53.8 | 38.6 | 7.6 |
| 10 | 55.2 | 39.3 | 5.5 |
| 11 | 64.6 | 31.2 | 4.2 |
| 12 | 59.5 | 34.7 | 5.7 |
| 13 | 60.7 | 34.7 | 4.6 |
| 14 | 55.6 | 37.0 | 7.4 |
| 15 | 69.1 | 26.2 | 4.7 |
| 16 | 67.6 | 27.5 | 5.0 |
| 18 | 65.8 | 27.6 | 6.6 |
| 19 | 59.1 | 33.3 | 7.6 |
| 20 | 64.7 | 28.5 | 6.8 |
| 21 | 58.7 | 33.0 | 8.3 |
| 22 | 62.8 | 30.2 | 7.0 |
| 23 | 58.3 | 35.1 | 6.6 |
| 24 | 58.9 | 34.0 | 7.1 |
| 25 | 60.4 | 31.8 | 7.8 |
| 26 | 61.1 | 31.9 | 7.0 |
| 27 | 61.8 | 29.8 | 8.4 |
| 28 | 64.4 | 29.0 | 6.6 |
| 29 | 64.3 | 29.2 | 6.5 |
| 30 | 64.2 | 28.5 | 7.4 |
| 32 | 56.4 | 37.2 | 6.5 |
| 34 | 59.1 | 33.0 | 7.9 |
| 35 | 53.3 | 37.0 | 9.6 |
| All | 58.3 | 34.7 | 7.0 |

**Source: NHSBT complementary survey to monitor the awareness of the law change (35 waves (2018-2022), average sample 1,420 with 5% ethnic minority per survey)**

**Supplementary file 6: Sources of information about the law change; NHSBT complementary survey to monitor the awareness of the law change (35 waves (2018-2022)**

**Supplementary Table 4: Test results for optimal number of population segments/subgroups by survey wave**

| **Classes** | **Log-likelihood** | **Number of parameters** | **AIC** | **CAIC** | **BIC** |
| --- | --- | --- | --- | --- | --- |
| Wave 1 |  |  |  |  |  |
| 2 | -2667.22 | 3 | 5340.432 | 5359.524 | 5356.524 |
| 3 | -2666.94 | 5 | 5343.875 | 5375.695 | 5370.695 |
| 4 | -2666.88 | 7 | 5347.753 | 5392.3 | 5385.3 |
| 5 | -2666.87 | 9 | 5351.732 | 5409.007 | 5400.007 |
| 6 | -2666.86 | 11 | 5355.725 | 5425.728 | 5414.728 |
| Wave 2 |  |  |  |  |  |
| 2 | -2393.45 | 3 | 4792.896 | 4811.803 | 4808.803 |
| 3 | -2393.16 | 5 | 4796.317 | 4827.83 | 4822.83 |
| 4 | -2393.12 | 7 | 4800.235 | 4844.353 | 4837.353 |
| 5 | -2393.1 | 9 | 4804.206 | 4860.928 | 4851.928 |
| 6 | -2393.58 | 11 | 4809.153 | 4878.481 | 4867.481 |
| Wave 3 |  |  |  |  |  |
| 2 | -1636.86 | 3 | 3279.721 | 3297.435 | 3294.435 |
| 3 | -1636.759 | 5 | 3283.519 | 3313.042 | 3308.042 |
| 4 | -1636.499 | 7 | 3286.999 | 3328.332 | 3321.332 |
| 5 | -1636.395 | 9 | 3290.79 | 3343.933 | 3334.933 |
| 6 | -1634.864 | 11 | 3291.727 | 3356.68 | 3345.68 |
| Wave 4 |  |  |  |  |  |
| 2 | -2452.222 | 3 | 4910.443 | 4929.361 | 4926.361 |
| 3 | -2450.434 | 5 | 4910.869 | 4942.398 | 4937.398 |
| 4 | -2450.308 | 7 | 4914.615 | 4958.756 | 4951.756 |
| 5 | -2450.185 | 9 | 4918.37 | 4975.123 | 4966.123 |
| 6 | -2450.17 | 11 | 4922.34 | 4991.704 | 4980.704 |
| Wave 5 |  |  |  |  |  |
| 2 | -2585.149 | 3 | 5176.299 | 5195.413 | 5192.413 |
| 3 | -2544.57 | 5 | 5099.14 | 5130.998 | 5125.998 |
| 4 | -2544.522 | 7 | 5103.044 | 5147.644 | 5140.644 |
| 5 | -2544.523 | 9 | 5107.047 | 5164.39 | 5155.39 |
| 6 | -2544.516 | 11 | 5111.031 | 5181.117 | 5170.117 |
| Wave 6 |  |  |  |  |  |
| 2 | -2966.223 | 3 | 5938.447 | 5957.968 | 5954.968 |
| 3 | -2950.774 | 5 | 5911.548 | 5944.084 | 5939.084 |
| 4 | -2950.736 | 7 | 5915.471 | 5961.021 | 5954.021 |
| 5 | -2950.721 | 9 | 5919.442 | 5978.007 | 5969.007 |
| 6 | -2950.692 | 11 | 5923.383 | 5994.962 | 5983.962 |
| Wave 7 |  |  |  |  |  |
| 2 | -2885.521 | 3 | 5777.042 | 5796.473 | 5793.473 |
| 3 | -2885.388 | 5 | 5780.776 | 5813.161 | 5808.161 |
| 4 | -2885.353 | 7 | 5784.707 | 5830.046 | 5823.046 |
| 5 | -2885.343 | 9 | 5788.685 | 5846.978 | 5837.978 |
| 6 | -2885.338 | 11 | 5792.676 | 5863.924 | 5852.924 |
| Wave 8 |  |  |  |  |  |
| 2 | -2937.16 | 3 | 5880.323 | 5899.778 | 5896.778 |
| 3 | -2937.12 | 5 | 5884.246 | 5916.67 | 5911.67 |
| 4 | -2937.11 | 7 | 5888.216 | 5933.611 | 5926.611 |
| 5 | -2937.23 | 9 | 5892.454 | 5950.819 | 5941.819 |
| 6 | -2937.13 | 11 | 5896.254 | 5967.588 | 5956.588 |
| Wave 9 |  |  |  |  |  |
| 2 | -2935.37 | 3 | 5876.731 | 5896.186 | 5893.186 |
| 3 | -2935.34 | 5 | 5880.685 | 5913.11 | 5908.11 |
| 4 | -2935.33 | 7 | 5884.664 | 5930.058 | 5923.058 |
| 5 | -2935.35 | 9 | 5888.69 | 5947.054 | 5938.054 |
| 6 | -2935.34 | 11 | 5892.67 | 5964.004 | 5953.004 |
|  |  |  |  |  |  |
| Wave 11 |  |  |  |  |  |
| 2 | -3862.19 | 3 | 7730.383 | 7750.608 | 7747.608 |
| 3 | -3862.16 | 5 | 7734.309 | 7768.017 | 7763.017 |
| 4 | -3862.17 | 7 | 7738.344 | 7785.535 | 7778.535 |
| 5 | -3862.15 | 9 | 7742.29 | 7802.964 | 7793.964 |
| 6 | -3862.15 | 11 | 7746.292 | 7820.449 | 7809.449 |
| wave 12 |  |  |  |  |  |
| 2 | -3306.56 | 3 | 6619.121 | 6638.865 | 6635.865 |
| 3 | -3306.52 | 5 | 6623.034 | 6655.94 | 6650.94 |
| 4 | -3306.5 | 7 | 6626.995 | 6673.063 | 6666.063 |
| 5 | -3306.5 | 9 | 6630.999 | 6690.23 | 6681.23 |
| 6 | -3306.49 | 11 | 6634.984 | 6707.377 | 6696.377 |

**Source: NHSBT Organ Donation Attitudinal Tracker survey data (2015-2022). Note: The determination of the optimal numbers of segments requires a balance assessment of the statistics presented in supplementary table 4. The work of Andrews and Currim (2003) demonstrated that the BIC and AIC statistics never under-fit but may sometimes over-fit the number of segments. Over-fitting the true number of segments produces larger parameter bias. We therefore used the minimum of either BIC or AIC to determine the number of segments for the respective waves. This is highlighted yellow in supplementary file Table 4.**

**Supplementary Table 5: Characteristics of respondents belonging to the four subgroups of deceased organ donation(row percentage)**

| **Characteristics** | **Sample**  **(n=2180)** | **Supportive donors** | **Sensitive donors** | **Ambivalent donors** | **Non donors** | **Chi2**  **p-value** |
| --- | --- | --- | --- | --- | --- | --- |
| **Share of subgroups in population** |  | **0.238** | **0.215** | **0.456** | **0.091** |  |
|  |  |  |  |  |  |  |
| **Average Age (years)***** | 2180 | 52 | 42 | 40 | 39 | 0.000 |
| **Willingness to donate***** |  |  |  |  |  |  |
| I would definitely donate **all** of my organs if possible | 752 | 76.6 | 0.9 | 21.5 | 0.9 | 0.000 |
| I would definitely donate **some** of my organs if possible | 317 | 0.6 | 4.1 | 91.8 | 3.5 |  |
| I would consider donating **all** of my organs | 283 | 0.0 | 16.6 | 71.7 | 11.7 |  |
| I would consider donating **some** of my organs | 272 | 0.0 | 18.4 | 65.8 | 15.8 |  |
| I don't know if I would donate my organs | 408 | 0.0 | 63.0 | 18.4 | 18.6 |  |
| I definitely wouldn't donate my organs | 148 | 0.0 | 64.2 | 17.6 | 18.2 |  |
| **Support of organ donation***** |  |  |  |  |  |  |
| I strongly support organ donation in principle | 899 | 57.0 | 1.2 | 39.3 | 2.6 | 0.000 |
| I support organ donation in principle | 690 | 9.6 | 6.5 | 76.4 | 7.5 |  |
| I neither support nor oppose organ donation in principle | 366 | 0.0 | 71.6 | 6.0 | 22.4 |  |
| I oppose organ donation in principle | 61 | 0.0 | 65.6 | 14.8 | 19.7 |  |
| I strongly oppose organ donation in principle | 72 | 0.0 | 56.9 | 31.9 | 11.1 |  |
| No response | 92 | 0.0 | 76.1 | 2.2 | 21.7 |  |
| **Aware of OD publicity***** |  |  |  |  |  |  |
| Yes | 905 | 28.2 | 19.0 | 49.5 | 3.3 | 0.000 |
| No | 1177 | 26.3 | 22.9 | 38.1 | 12.8 |  |
| Don't know | 98 | 14.3 | 28.6 | 40.8 | 16.3 |  |
| **Awareness of organ donor register***** |  |  |  |  |  |  |
| Yes | 1746 | 30.9 | 19.1 | 45.7 | 4.4 | 0.000 |
| No | 330 | 7.9 | 30.9 | 32.4 | 28.8 |  |
| Don't Know | 104 | 11.5 | 32.7 | 30.8 | 25.0 |  |
| **Registered decision on organ donor register***** |  |  |  |  |  |  |
| Yes, I have registered my decision | 793 | 48.3 | 12.4 | 37.0 | 2.4 | 0.000 |
| No, I don’t think so | 877 | 13.5 | 24.7 | 53.3 | 8.6 |  |
| Not sure/don’t know | 203 | 25.1 | 26.6 | 42.9 | 5.4 |  |
| No response | 307 | 8.5 | 32.6 | 29.0 | 30.0 |  |
| **Whether spoken to anyone about organ donation ***** |  |  |  |  |  |  |
| Yes | 1032 | 40.4 | 12.5 | 43.1 | 4.0 | 0.000 |
| No | 1066 | 13.7 | 29.4 | 43.3 | 13.7 |  |
| Don't know | 82 | 18.3 | 32.9 | 36.6 | 12.2 |  |
| **Specific discussion about whether or not to donate organ with close family or partner***** |  |  |  |  |  |  |
| Yes – have told them about my decision around organ donation | 766 | 50.0 | 9.7 | 38.6 | 1.7 | 0.000 |
| No – have not told them about my decision around organ donation | 218 | 9.2 | 20.2 | 58.7 | 11.9 |  |
| Don't know | 48 | 29.2 | 22.9 | 43.8 | 4.2 |  |
| No response | 1148 | 14.0 | 29.6 | 42.8 | 13.6 |  |
| **OD decisions told close family or partner***** |  |  |  |  |  |  |
| I told them that I want my organs to be donated | 663 | 57.6 | 2.7 | 38.8 | 0.9 | 0.000 |
| I told them that I do not want my organs to be donated | 96 | 0.0 | 54.2 | 38.5 | 7.3 |  |
| Don't know | 7 | 14.3 | 57.1 | 28.6 | 0.0 |  |
| **Specifically told close family or partner that you want them to support your registered decision****i*** |  |  |  |  |  |  |
| Yes – have told them that I want them to support my decision | 628 | 83.8 | 77.1 | 82.7 | 84.6 | 0.000 |
| No – have not told them that I want them to support my decision | 101 | 12.6 | 20.0 | 12.6 | 15.4 |  |
| Don't know | 30 | 3.7 | 2.9 | 4.8 | 0.0 |  |
| **Sex***** |  |  |  |  |  |  |
| Male | 979 | 50.4 | 28.1 | 42.3 | 81.2 | 0.000 |
| Female | 1198 | 49.5 | 71.9 | 57.6 | 18.3 |  |
| Other | 3 | 0.2 | 0.0 | 0.1 | 0.5 |  |
| **Region***** |  |  |  |  |  |  |
| North West England | 292 | 34.3 | 12.0 | 35.6 | 18.2 | 0.000 |
| North East England | 115 | 48.7 | 7.0 | 39.1 | 5.2 |  |
| Yorkshire and the Hum | 186 | 37.1 | 12.4 | 33.9 | 16.7 |  |
| West Midlands | 239 | 26.8 | 27.2 | 33.1 | 13.0 |  |
| East Midlands | 197 | 33.0 | 14.7 | 32.5 | 19.8 |  |
| East Anglia | 182 | 23.1 | 20.9 | 55.5 | 0.6 |  |
| London | 481 | 3.3 | 33.7 | 60.5 | 2.5 |  |
| South East England (excluding London) | 310 | 32.6 | 20.0 | 40.3 | 7.1 |  |
| South West England | 178 | 36.5 | 26.4 | 36.0 | 1.1 |  |
| **Ethnic origin***** |  |  |  |  |  |  |
| Other | 573 | 4.5 | 32.8 | 42.9 | 19.7 | 0.000 |
| White | 1607 | 34.4 | 17.5 | 42.9 | 5.2 |  |
| **Faith base***** |  |  |  |  |  |  |
| Christianity | 1007 | 25.8 | 16.5 | 46.7 | 11.0 | 0.000 |
| Islam | 213 | 0.0 | 55.4 | 31.9 | 12.7 |  |
| Hinduism | 70 | 12.9 | 14.3 | 54.3 | 18.6 |  |
| Sikhism | 30 | 16.7 | 26.7 | 43.3 | 13.3 |  |
| Buddhism | 15 | 33.3 | 33.3 | 33.3 | 0.0 |  |
| Judaism | 15 | 26.7 | 26.7 | 46.7 | 0.0 |  |
| Other | 41 | 34.2 | 26.8 | 31.7 | 7.3 |  |
| Prefer not to say | 789 | 35.6 | 18.6 | 40.8 | 4.9 |  |

**Note: ***Pearson’s Chi-square test showing significant difference among subgroup at 1% level (p<0.01); *i* sample was restricted to those who have registered a decision on the organ donor register; Source: NHSBT Organ Donation Attitudinal Tracker survey data (2015-2022)**

**Supplementary Table 6: Characteristics of interviewees**

| **Category** | **General public (n=30)** |
| --- | --- |
| Gender |  |
| Male | 11 |
| Female | 19 |
|  |  |
| Ethnicity |  |
| Black African/American | 6 |
| Asian | 16 |
| White | 8 |
| Mixed race |  |
| Other (White, Chinese) |  |
|  |  |
| Religion |  |
| Christian | 6 |
| Muslim | 18 |
| Other (Jewish, Sikh, Hindu, no religion) | 6 |
|  |  |
| Supported the organ donation decision |  |
| Yes |  |
| No |  |
| Awareness of the law change |  |
| Yes | 24 |
| No | 6 |
|  |  |
| Supportive of the chance |  |
| Yes | 27 |
| No | 3 |
|  |  |
| Decision on organ donor register |  |
| Opted-in | 6 |
| Opted out | 10 |
| Uncertain | 14 |

**Source: Qualitative survey data**
